# Increasing Lipid Nanoparticle mRNA Copy Number Improves Transfection Potency

**DOI:** 10.1002/adma.75174

**Published:** 2026-09-27

**Authors:** Yao Zhang, Jerry Leung, Judy Cheng, Arash Momeni, Albert Kamanzi, Ariadne Tuckmantel Bido, Suiyang Liao, Emma Kang, Colton Strong, Christian J. Kastrup, Sabrina Leslie, Miffy Hok Yan Cheng, Pieter R. Cullis

**Affiliations:** ^1^ Department of Biochemistry and Molecular Biology University of British Columbia Vancouver British Columbia Canada; ^2^ School of Biomedical Engineering University of British Columbia Vancouver British Columbia Canada; ^3^ Michael Smith Laboratories University of British Columbia Vancouver British Columbia Canada; ^4^ Department of Psychiatry Djavad Mowafaghian Centre for Brain Health University of British Columbia Vancouver British Columbia Canada; ^5^ Department of Surgery Medical College of Wisconsin Milwaukee Wisconsin USA; ^6^ Versiti Blood Research Institute Milwaukee Wisconsin USA; ^7^ Department of Physics and Astronomy University of British Columbia Vancouver British Columbia Canada; ^8^ Faculty of Pharmaceutical Sciences University of British Columbia Vancouver British Columbia Canada

**Keywords:** drug delivery, gene therapy, lipid nanoparticles, mRNA loading, nanomedicine

## Abstract

Lipid nanoparticles (LNPs) are the leading platform for delivery of RNA‐based therapeutics. There is considerable interest in optimizing the transfection potency of these systems. Standard formulation protocols involve mixing lipids in ethanol with a buffer containing mRNA at pH 4, followed by dialysis against a pH 7.4 saline buffer to remove ethanol and raise the pH. The dialysis process results in fusion of mRNA‐lipid complexes and small vesicles formed at pH 4, driven by two processes: Conversion of the ionizable lipid from the positively charged bilayer form to the neutral oil form and fusion stimulated by higher ionic strengths. Here we explored the consequences of a two‐step dialysis process involving formulating at pH 4, 25 mM NaOAc, and then inducing fusion at pH 4 by dialyzing against a higher ionic strength buffer while maintaining the ethanol concentration at 25% (v/v). This was followed by dialysis against PBS to raise the pH. We show that the two‐step protocol results in increased mRNA loading per LNP, a decreased proportion of “empties” as compared to the standard process, and improved transfection potencies in vitro and in vivo when compared to LNP mRNA formed from the traditional one‐step buffer exchange process.

## Graphical abstract

As lipid nanoparticle (LNP) systems are the leading platform for in vivo nucleic acid delivery, improving transfection potency of these systems is of considerable interest. In this work, we explored a post‐mixing formulation method involving a two‐step buffer exchange that increased the number of mRNA copies within each LNP while reducing the number of empty particles, resulting in transfection improvements in vitro and in vivo.

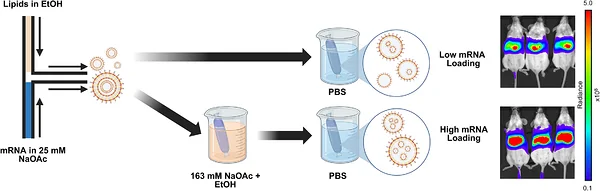

## 1 Introduction

Lipid nanoparticles (LNPs) are increasingly the leading platform for in vivo delivery of nucleic acid cargo [1]. Clinical successes include the regulatory approval of Onpattro in 2018 (the first RNAi therapeutic) [2] and the approval of the LNP mRNA COVID‐19 vaccines in 2020 [3, 4]. There are now over 400 LNP mRNA therapeutics in development, with over 100 having reached the clinic [1, 5]. Basic research efforts are now focused on extending the utility of LNP RNA systems to transfect extrahepatic tissues and on increasing the overall transfection potency of these systems.

There is confusion in the field as to when LNP RNA particles are actually formed during the formulation process. Some assume that the particles form at pH 4 when the lipids in ethanol (EtOH) are mixed with nucleic acid cargos dissolved in a pH 4 aqueous buffer. However, this is not the case. At pH 4, the ionizable lipids are protonated (positively charged) and able to adopt a bilayer structure in an aqueous environment. Given that bilayer lipids dissolved in EtOH form very small vesicles when dispersed in an aqueous medium [6], the ionizable lipids, in combination with the “helper” lipids (e.g., distearoylphosphatidylcholine (DSPC) and cholesterol), form small positively charged bilayer structures [7]. At pH 4, a proportion of these combine with the negatively charged nucleic acid cargo to form complexes. Subsequently, as the preparation is dialyzed against PBS to eliminate EtOH and raise the pH to physiological values, the ionizable lipids adopt the neutral form, leading to the small vesicles fusing with each other and with the nucleic acid‐containing complexes [7]. The fusion process ends when the concentration of PEG‐lipid on the exterior monolayer inhibits further fusion.

The process outlined above potentially leads to a heterogeneous LNP mixture. LNP RNA systems with varying RNA payloads and lipid compositions can be formed, as well as empty LNP systems. Techniques that encourage formation of more homogeneous LNP RNA systems are required. In this regard, early studies on LNP‐nucleic acid systems showed that lipid components are able to flip‐flop rapidly across bilayers and exchange rapidly between LNP in the presence of 25%–40% EtOH (v/v) [8]. Further, more recent studies have shown that fusion can be induced early in the formulation process by formulating in pH 4 buffers with high ionic strengths [9], resulting in improved transfection properties. Here we explore whether more homogeneous and more potent LNP RNA systems can be achieved by using a two‐step dialysis process. The first dialysis is aimed at inducing fusion and also improving homogeneity by using a reasonably high ionic strength, pH 4 dialysis medium containing high levels of EtOH. The second dialysis is against PBS to remove EtOH and raise the pH. We show that the resulting LNP mRNA systems exhibit a higher mRNA copy number per LNP, a reduced proportion of empty LNP, and enhanced transfection potency both in vitro and in vivo.

## 2 Results

### 2.1 Identification of Optimized Dialysis Procedures

LNP consisting of norMC3/DSPC/cholesterol/PEG‐DMG (50/10/38.5/1.5 mol%, respectively; the “Onpattro‐like” lipid composition) formulated using a T‐junction mixer in a 25 mM NaOAc pH 4 medium and containing mRNA encoding firefly luciferase (FLuc) (N/P = 6) were taken as the starting material. The next step in the formulation process is typically dialysis against PBS to raise the pH and remove EtOH. As noted, raising the pH converts the ionizable lipid to the neutral form, destabilizing the vesicles formed at pH 4 and inducing fusion [7, 10]. Here we use the first dialysis to induce fusion at pH 4 rather than raising the pH. Previous work has shown that pH 4 media with high ionic strength NaOAc can induce fusion between LNPs with the Onpattro‐like composition [9, 10]. We chose a pH 4 buffer containing 163 mM NaOAc as a suitable medium, as it induced essentially complete fusion of LNP systems consisting of norMC3/DSPC/Cholesterol/PEG‐DMG (50/10/38.5/1.5 mol%, respectively) within 6 h (see Figure S1).

Two types of formulations were generated for further studies using the protocol summarized in Figure 1A. The first (control) formulation (cLNP mRNA) used the standard one‐step dialysis protocol against PBS following T‐tube mixing with 25 mM NaOAc at pH 4. The second two‐step formulation involving dialysis in the presence of EtOH (eLNP mRNA) was generated by first formulating in the presence of 25 mM NaOAc at pH 4 and then dialyzing against a pH 4, 163 mM NaOAc buffer containing EtOH (0%–35% (v/v)), followed by a second dialysis against PBS. The eLNP mRNA formulation gave rise to somewhat larger systems than the cLNP mRNA as detected by dynamic light scattering (DLS; Figure 1B), increasing from a diameter of ∼50 nm for the cLNP to ∼75 nm for the eLNP formulations dialyzed in the presence of 25% (v/v) EtOH. It may be noted that all formulations resulted in PDI values below 0.1 (Figure 1C) with high encapsulation efficiencies (Figure 1D). Higher EtOH contents (35%) gave rise to reduced N/P values (Figure 1E), indicating loss of lipids (Figure S2), and resulted in larger particle sizes (Figure 1B,F and Figure S3). The eLNP mRNA systems dialyzed against 25% (v/v) EtOH were used in subsequent work.

**FIGURE 1:**
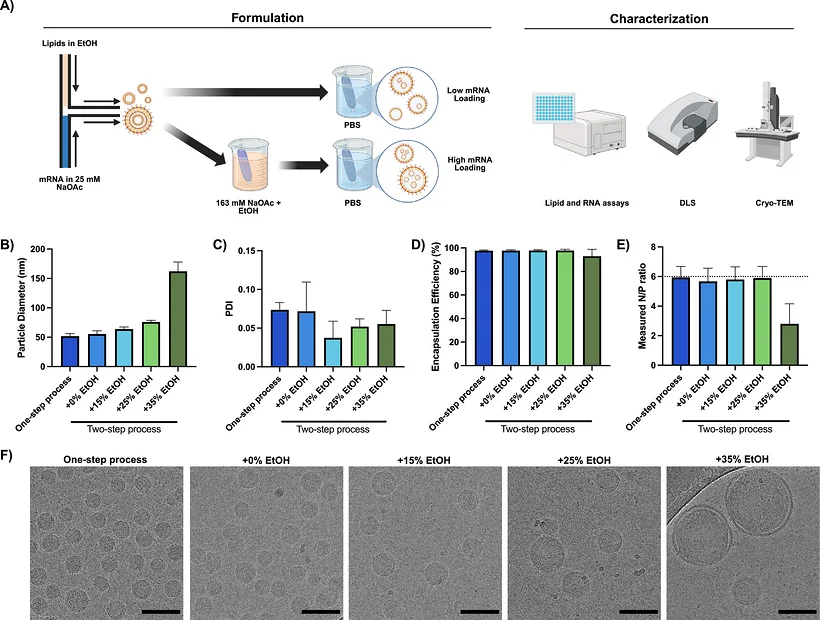
LNP mRNA formulations produced from the two‐step buffer exchange method result in desirable physiochemical properties with larger particle sizes compared to the standard one‐step buffer exchange process. (A) LNP mRNA (FLuc; *N/P* = 6) was formulated through a T‐junction mixer with a lipid composition of norMC3/DSPC/Cholesterol/PEG‐DMG (50/10/38.5/1.5 mol%, respectively) and was subjected to a one‐step (PBS dialysis) or two‐step (163 mM NaOAc + 0%/15%/25%/35% EtOH dialysis prior to PBS dialysis) buffer exchange. Standard LNP characterization methods were subsequently performed, including the (B) particle size (Z‐average) (mean ± S.D., *n* = 4), (C) polydispersity index (PDI) (mean ± S.D., *n* = 4), (D) encapsulation efficiencies (mean ± S.D., *n* = 4), and (E) measured *N/P* ratios compared to the theoretical target (*N/P* = 6) (mean ± S.D., *n* = 3). (F) Cryo‐TEM micrographs of the different LNP mRNA systems. Scale bar = 100 nm.

It should be noted that this fusion process does not depend on encapsulated cargo, as empty LNP formulations that were subjected to the two‐step buffer exchange process exhibited similar results (Figure S4). To further demonstrate LNP‐LNP fusion in the two‐step buffer exchange process is induced by the pH 4 163 mM NaOAc + EtOH buffer, the particle size at pH 4 of empty LNP (Figure S5) and LNP mRNA formulations (Figure S6) also increased as the EtOH content was raised. All formulations except for particles dialyzed against 35% (v/v) EtOH displayed a predominately oil core morphology following dialysis against PBS (Figure 1F; Figures S7 and S8).

### 2.2 LNP mRNA Systems Formulated Through the Two‐Step Buffer Exchange Process Result in More Copies of mRNA per LNP

The objective of this study was to improve the homogeneity of LNP mRNA systems by introducing the EtOH‐induced equilibration step at pH 4, which may be expected to result in exchange of lipids between LNPs to achieve equilibrium [8]. There is also the possibility that the mRNA loading of LNP could be enhanced due to fusion of complexes formed at pH 4. We have recently demonstrated that convex lens‐induced confinement (CLiC) imaging can be used to detect loaded versus unloaded LNP as well as the number of copies of mRNA per LNP [11, 12]. We therefore employed this technique to measure the proportion of empty LNP in an LNP mRNA preparation and the number of mRNA copies per LNP by incorporating DiO (a lipophilic dye) into the LNP and encapsulating Cy5‐FLuc mRNA. After T‐tube mixing, the particles were subjected to either the normal dialysis buffer exchange process using PBS to produce cLNP mRNA or the eLNP mRNA formulation employing the two‐step dialysis process. Single‐particle characterization was then performed on the cLNP mRNA and eLNP mRNA formulations employing CLiC imaging. Loaded LNP were distinguished from empty LNP by detecting the co‐localization of the mRNA Cy5 and LNP DiO fluorophores in a given LNP. Additionally, mRNA loading estimates were derived by normalizing the measured LNP mRNA Cy5 signal with the Cy5 signal of free mRNA. Finally, the hydrodynamic radius of an LNP was determined by measuring its rate of lateral diffusion in aqueous media and then deriving the radius employing a modified Stokes‐Einstein equation [11, 13].

As shown in Figure 2, the cLNP exhibited 83% loading as reported by the CLiC analysis whereas the eLNP exhibited 91% loading. Further, the cLNP only contained ∼2 copies of mRNA per LNP (Figure 2A) as opposed to the eLNP which contained an average of ∼6 copies of mRNA per LNP (Figure 2B). The CLiC analysis also revealed a smaller average particle size for both the mRNA loaded and empty LNPs of the cLNP mRNA formulation (*r*_loaded_ = 42.2 ± 0.3 nm, *r*_empty_ = 37.3 ± 0.5 nm) versus the eLNP system (*r*_loaded_ = 63.1 ± 0.5 nm, *r*_empty_ = 42.1 ± 1.0 nm) (Figure 2A,B), in agreement with DLS data. The increased mRNA loading for the eLNP mRNA formulation is consistent with the increase in size as detected by DLS and CLiC. This presumably arises due to fusion of vesicles and vesicle‐mRNA complexes formed at pH 4 together with redistribution of lipid due to the presence of 25% EtOH in the dialysis medium. The decrease in the proportion of small empty LNP likely arises due to an Ostwald ripening effect that promotes lipid transfer to the larger LNP systems.

**FIGURE 2:**
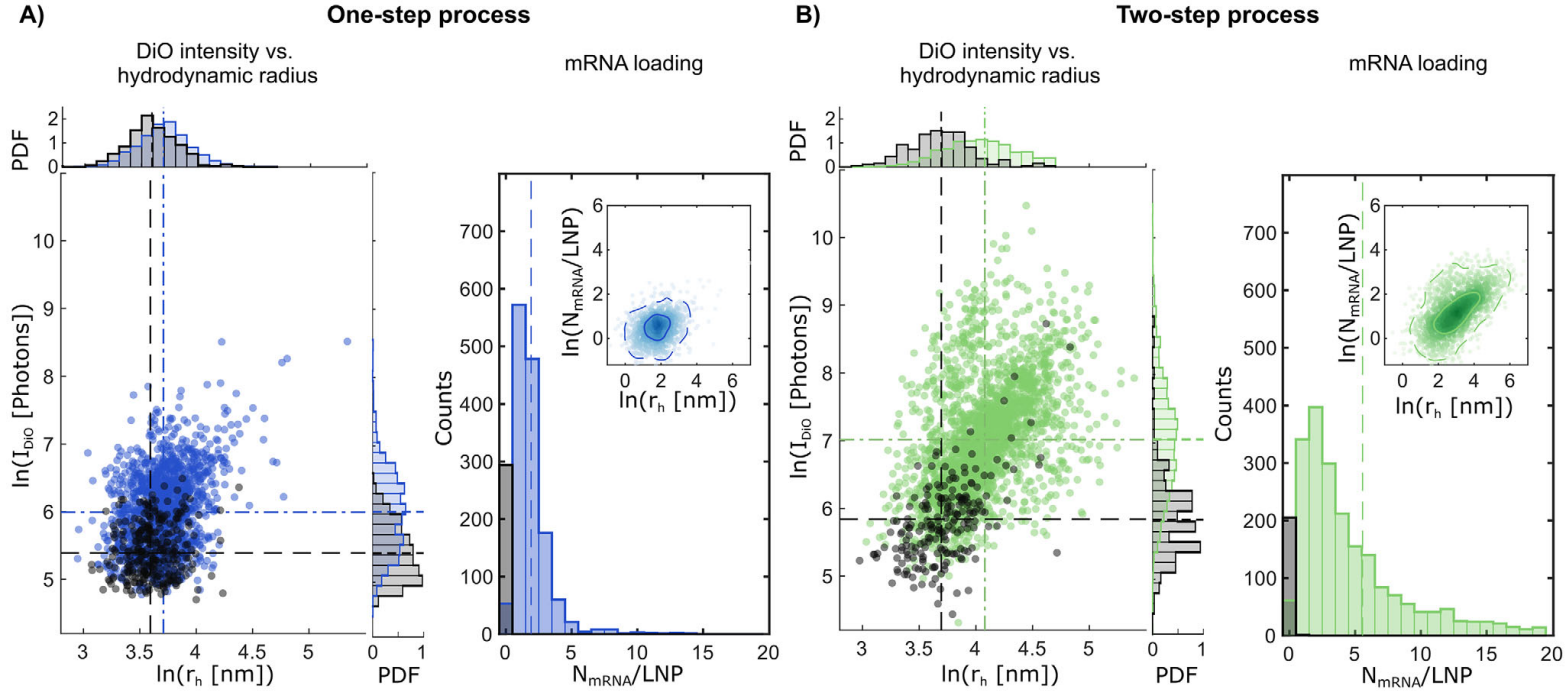
LNP mRNA formulations subjected to the two‐step buffer exchange process result in more mRNA copies per LNP and a lower proportion of empty LNPs compared to the standard one‐step buffer exchange procedure. LNP mRNA (Cy5‐FLuc; *N/P* = 6) were formulated through a T‐junction mixer with a lipid composition of norMC3/DSPC/Cholesterol/PEG‐DMG/DiO (50/10/38.5/1.5/1 mol/mol, respectively) and were subjected to a one‐step (PBS dialysis) or two‐step (163 mM NaOAc + 25% EtOH dialysis prior to PBS dialysis) buffer exchange. LNP mRNA formulations were then characterized through single‐particle CLiC imaging. (A, B) Single particle characterization results for (A) the one‐step buffer exchange process (*r*_loaded_ = 42.2 ± 0.3 nm, *r*_empty_ = 37.3 ± 0.5 nm; loading fraction = 83%, Mean_mRNA/LNP_ = 2, Median_mRNA/LNP_ = 2) and (B) the two‐step buffer exchange process (*r*_loaded_ = 63.1 ± 0.5 nm, *r*_empty_ = 42.1 ± 1.0 nm; loading fraction = 91%, Mean_mRNA/LNP_ = 6, Median_mRNA/LNP_ = 4). The left plot of each subfigure depicts the DiO Intensity versus particle size r (radius) for loaded (colored dots) and empty (black dots) LNPs, while the right plot depicts the distribution of the estimated copies of mRNA/LNP for loaded (colored bars) and empty (black bars) LNPs. All dotted lines represent the arithmetic mean. The inset figure shows the mean copies of mRNA per LNP (N_mRNA/LNP_) versus particle size *r* for loaded LNPs.

The loading procedure used to load RNA into LNP employing ionizable cationic lipids can also be used to load other negatively charged entities such as citrate‐coated gold nanoparticles (GNP) [14]. To visually demonstrate the equilibration process engendered by incubating in the pH 4 buffer containing EtOH, we investigated the effects of the two‐step buffer exchange process on loading GNP. The electron‐dense GNP can be readily visualized in LNP systems by employing cryo‐TEM. LNP GNP systems with the Onpattro‐based composition were generated in which 5 nm negatively charged GNPs (2.2E+13 GNP/µmol lipid) were substituted for mRNA using the one‐step and two‐step loading procedures. As shown in Figure S9A, the two‐step procedure resulted in improved GNP loading and a more homogeneous distribution of GNP. Quantitative analysis of 200 LNP from cryo‐TEM micrographs revealed that LNP GNP formulated through the two‐step buffer exchange process resulted in a dramatic reduction of empty LNPs and increased numbers of GNP in the loaded LNP (92% loaded; 4.5 GNP per LNP) compared to particles generated from the one‐step buffer exchange procedure (55% loaded; 1.6 GNP per LNP) (Figure S9B). Similar size increases following the two‐step dialysis procedure as observed for the mRNA‐containing systems were also apparent. The two‐step buffer exchange process resulted in a larger particle size (∼70 nm) compared to particles that had undergone the one‐step buffer exchange process (∼45 nm) (Figure S9C). The particle diameter increased with the number of GNP/LNP encapsulated (Figure S9D,E).

### 2.3 LNP mRNA Systems Formulated Through the Two‐Step Buffer Exchange Process Exhibit Improved Transfection Potency In Vitro

It is of obvious interest to determine the influence of the increased mRNA loading noted for the eLNP system on transfection potency. The in vitro transfection potencies were examined using Huh7 cells. As shown in Figure 3A–C (corresponding to a mRNA dose of 0.3, 1.0, and 3.0 µg/mL, respectively), eLNP mRNA formulations that were subjected to the two‐step buffer exchange procedure were significantly more transfection potent than the cLNP mRNA that used the traditional one‐step buffer exchange process. At a dose of 1.0 µg/mL mRNA, the eLNP mRNA systems exhibited a ∼16‐fold increase in luminescence as compared to the cLNP mRNA formulation. A similar improvement in transfection potency for eLNP systems can be observed for lower mRNA doses (Figure S10) without indication of cytotoxicity (Figure S11). Additionally, the benefits of the two‐step buffer exchange process also extend to LNP mRNA systems formulated with 25 mM sodium citrate buffer (Figure S12).

**FIGURE 3:**
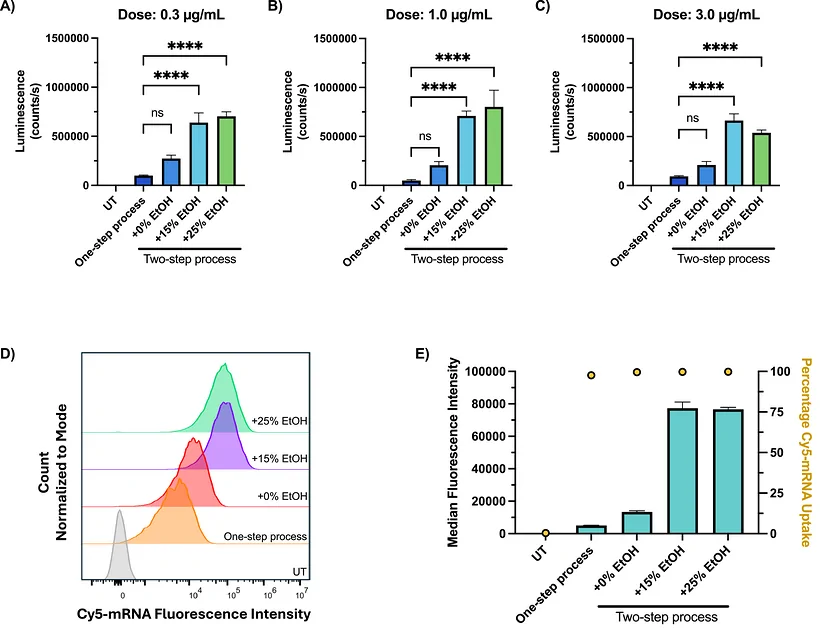
LNP mRNA produced from the two‐step buffer exchange process improves in vitro transfection and increases mRNA delivery to cells compared to LNP mRNA produced from a one‐step buffer exchange. LNP mRNA (FLuc; *N/P* = 6) was formulated with a lipid composition consisting of norMC3/DSPC/Cholesterol/PEG‐DMG (50/10/38.5/1.5 mol%, respectively) and was subjected to a one‐step (PBS dialysis) or two‐step (163 mM NaOAc + 0%/15%/25% EtOH dialysis prior to PBS dialysis) buffer exchange. (A–C) Luminescence readout of Huh7 cells after 24 h incubation with LNP mRNA formulations at an mRNA dose of (A) 0.3 µg/mL, (B) 1.0 µg/mL, and (C) 3.0 µg/mL (mean ± S.E.M., *n* = 6). Data was analyzed by a one‐way ANOVA; ns = nonsignificant, ****p < 0.0001. (D) Representative flow cytometry plots of Huh7 cells after 24 h incubation with LNP mRNA formulations at an mRNA dose of 0.3 µg/mL and (E) corresponding quantification of Cy5 median fluorescence intensity (bars, left y‐axis) and percentage of Huh7 cells positive for Cy5‐mRNA (yellow circles, right y‐axis) (mean ± S.E.M., *n* = 3). UT = untreated cells.

It is likely that this improved transfection potency is related to the higher mRNA loading of eLNP formulations which can potentially deliver more mRNA copies into cells. To test this, we encapsulated Cy5‐labeled mRNA into the LNPs to enable a flow cytometry analysis. As shown in Figure 3D,E, the median fluorescence intensity of Cy5 was substantially greater for the eLNP mRNA formulations that were subjected to an initial dialysis against either a pH 4 163 mM NaOAc + 15% EtOH buffer (15.4‐fold increase) or a pH 4 163 mM NaOAc + 25% EtOH buffer (15.2‐fold increase) as compared to the cLNP mRNA formulation. It would appear that the improved transfection potency observed in formulations prepared from the two‐step buffer exchange procedure arises, at least in part, from increased intracellular mRNA delivery.

### 2.4 LNP mRNA Systems Formulated Through the Two‐Step Buffer Exchange Process Improve Transfection Potency for Cells Ex Vivo

Considerable efforts have been made to optimize LNP mRNA systems for ex vivo applications such as transfection of platelets [15, 16]. In order to determine whether the highly loaded eLNP have benefits in this ex vivo context we examined the transfection properties of eLNP mRNA systems in human donor platelets. We used a previously optimized lipid formulation for platelet transfection that contained the proprietary ionizable lipid NTX‐001 [16]. LNPs consisting of NTX‐001/POPC/Cholesterol/PEG‐DMG (50/10/38.5/1.5 mol%, respectively) were formulated encapsulating mRNA encoding for NanoLuc luciferase. LNP formed after T‐tube mixing in 25 mM NaOAc were dialyzed against pH 7.4 PBS (one‐step buffer exchange) or against pH 4 163 mM NaOAc + 25% EtOH prior to final PBS dialysis (two‐step buffer exchange). The eLNP mRNA systems using the two‐step buffer exchange method resulted in larger particles compared to formulations generated from the one‐step buffer exchange process (Figure 4A–C). As shown in Figure 4D the transfection potency of the eLNP mRNA was substantially improved without inducing platelet activation (Figure S13) at both 4 and 24 h post‐treatment as compared to the cLNP mRNA formulation. This improvement could be due to the increased presence of blebs observed in LNP mRNA systems formulated from the two‐step buffer exchange process (Figures S14 and S15) [9]. Despite examining different model organisms, these results indicate that the improvement in transfection potency for eLNP mRNA systems is potentially generalizable to systems that contain different ionizable cationic lipids.

**FIGURE 4:**
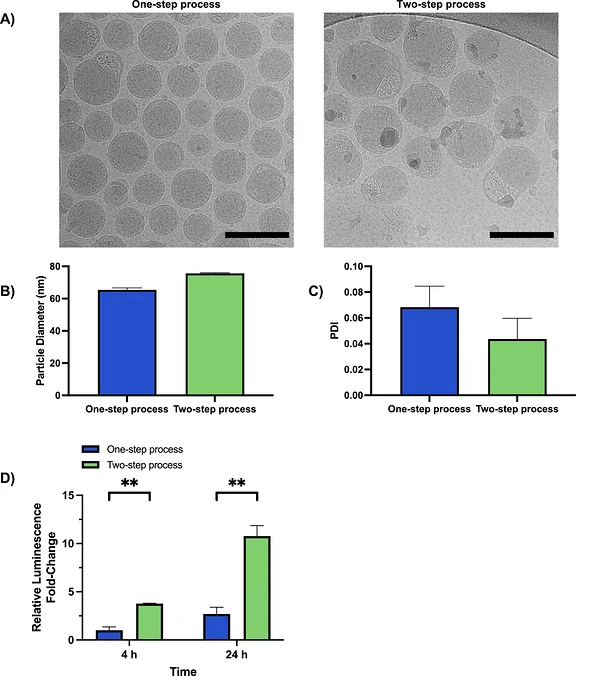
LNP mRNA produced from the two‐step buffer exchange process improves ex vivo transfection of platelets compared to LNP mRNA produced from a one‐step buffer exchange. LNP mRNA (nLuc; *N/P* = 6) were formulated with a lipid composition consisting of NTX‐001/POPC/Cholesterol/PEG‐DMG (50/10/38.5/1.5 mol%, respectively) and were subjected to a one‐step (PBS dialysis) or two‐step (163 mM NaOAc + 25% EtOH dialysis prior to PBS dialysis) buffer exchange. Platelets were transfected at a concentration of 250 × 10^6^ platelets/mL, and at an mRNA dose of 12 µg/mL. Luminescence was assayed at 4 and 24 h. (A) Cryo‐TEM micrographs of LNP mRNA undergoing the two different buffer exchange processes. Scale bar = 100 nm. (B) Particle size (*Z*‐average) (mean ± S.D., *n* = 3) and (C) PDI measurements of the LNP mRNA formulations determined through DLS (mean ± S.D., *n* = 3). (D) Relative luminescence fold‐change at 4 and 24 h normalized to the 4 h PBS dialysis readout (mean ± S.E.M., *n* = 3). Data were analyzed through a two‐tailed unpaired *t*‐test, **p < 0.01.

### 2.5 LNP mRNA Systems Formulated Through the Two‐Step Buffer Exchange Process Improve Transfection Potency In Vivo

As a final step in this study, we examined the benefits of the eLNP mRNA formulation for in vivo transfection. LNP composed of norMC3/DSPC/Cholesterol/PEG‐DMG (50/10/38.5/1.5 mol%, respectively) were formulated with FLuc mRNA and were subjected to either the two‐step buffer exchange or with the one‐step buffer exchange before intravenous (i.v.) administration into CD‐1 mice. As shown in Figure 5A,B, the eLNP mRNA systems resulted in improved transfection compared to the cLNP mRNA system at both 4 and 24 h post‐injection, with a 1.6‐fold and 3.1‐fold increase in luminescence, respectively (Figure 5C,D).

**FIGURE 5:**
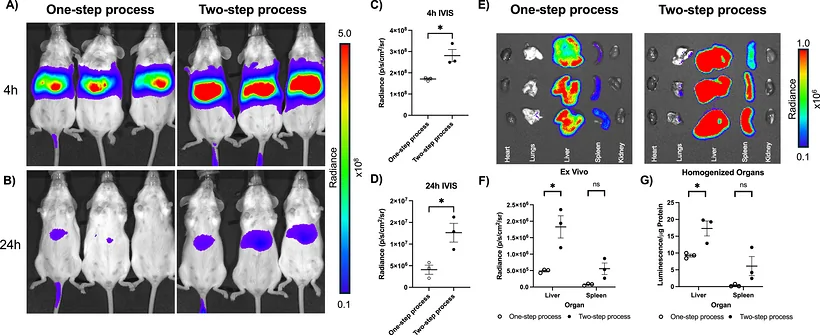
LNP mRNA produced from the two‐step buffer exchange process improves in vivo transfection following i.v. administration compared to LNP mRNA produced from a one‐step buffer exchange. LNP mRNA (FLuc; *N/P* = 6) was formulated with a lipid composition consisting of norMC3/DSPC/Cholesterol/PEG‐DMG (50/10/38.5/1.5 mol%, respectively) and was subjected to a one‐step (PBS dialysis) or two‐step (163 mM NaOAc + 25% EtOH dialysis prior to PBS dialysis) buffer exchange. (A–D) LNP mRNA was administered i.v. at an mRNA dose of 0.2 mg/kg to CD‐1 mice. IVIS bioluminescence imaging was performed at (A) 4 h and (B) 24 h post‐injection, with the corresponding radiance shown in (C) and (D), respectively (mean ± S.E.M., *n* = 3). (E) Ex vivo bioluminescence in the heart, lungs, liver, spleen, and kidney. (F) Ex vivo radiance quantification of liver and spleen (mean ± S.E.M., *n* = 3). (G) Luminescence of the homogenized liver and spleen normalized to the total protein content (mean ± S.E.M., *n* = 3). All data were analyzed through a two‐tailed unpaired *t*‐test; ns = nonsignificant, *p < 0.05.

At 24 h post‐injection, the mice were euthanized, and heart, lungs, liver, spleen, and kidney were excised for imaging ex vivo via IVIS. As shown in Figure 5E,F, the ex vivo signal observed in the liver was significantly (3.8‐fold) higher for the eLNP mRNA systems as compared to the luminescence observed for the cLNP mRNA formulation. A similar trend in liver and spleen luminescence signal was observed following organ homogenization (Figure 5G).

We further examined whether formulations made with the two‐step buffer exchange procedure can improve transfection potency following local administration. The eLNP mRNA and cLNP mRNA formulations were therefore administered via intramuscular administration (Figure S16) and demonstrated improvements in muscle transfection, though not statistically significant.

## 3 Discussion

We have shown that LNP mRNA systems formulated with a two‐step buffer exchange process involving a higher ionic strength pH 4 buffer and up to 25% EtOH content display greater mRNA loading and improved transfection potencies. Prior studies [17, 18] have demonstrated that payload capacity could be increased by reducing PEG‐lipid or by decreasing the *N/P* ratio; however, reducing the mol% of PEG‐lipid can lead to aggregation and particle instability during storage, while increasing the amount of mRNA‐to‐ionizable lipid can decrease encapsulation efficiencies [19]. Three areas warranting further discussion concern the ability of the pH 4 dialysis step to reduce the proportion of empty LNP, the mechanism whereby the two‐step procedure results in increased mRNA copy numbers per LNP, and finally why LNP mRNA systems containing higher copy numbers of mRNA exhibit more potent transfection properties.

The reduction in the proportion of empty LNP from 17% to 9% as shown in Figure 2 following the ethanol dialysis step addresses an important problem for LNP mRNA formulations, as empty systems do not contribute to transfection potency but will contribute to any toxicity associated with the ionizable cationic lipid content. This feature is more pronounced for the LNP GNP systems (Figure S9), where the proportion of empty LNP decreases from 45% to 8% as a result of dialysis in the presence of 25% EtOH. The proportion of empty LNP would be expected to decrease given the increase in diameter and decrease in particle concentration for eLNP mRNA systems (Figure S17). As shown in Figure 1B, the two‐step procedure results in an increase in diameter from 52 to 76 nm as compared to the one‐step process. Since the particle volume scales as the cube of the LNP diameter, the mean number of 52 nm cLNP contributing to a 76 nm eLNP can be approximated by *n*, where $n={\left(\frac{eLNP\;diameter}{cLNP\;diameter}\right)}^{3}$. Therefore, it takes ∼3.1 cLNPs to make an eLNP particle, indicating that the concentration of particles in the eLNP formulation should be three‐fold reduced as compared to the cLNP formulation. This is in approximate agreement with DLS particle concentration measurements (Figure S17) where the cLNP formulation is 3.7‐fold more concentrated than eLNP particles.

A potential mechanism of the two‐step process leading to enhanced mRNA copy numbers per LNP is shown in Figure 6. Fusion between mRNA‐cationic vesicle complexes leads to increased copy number per LNP whereas fusion between empty vesicles and complexes results in a reduction in the proportion of empty LNP. It may be noted that the driver of fusion is likely to be different for the one‐step process as compared to the two‐step process. In the one‐step dialysis process against PBS, two fusion stimuli are experienced at once‐the fusogenic effect of higher ionic strengths and the effect of raising the pH. Higher pH values destabilize the small bilayer vesicles by converting the ionizable lipid to the neutral form which phase separates from the bilayer into an oil core [7]. Which of these effects predominate in the one‐step process is not established. Previous work suggests that pH‐induced fusion proceeds more rapidly than ionic strength induced fusion (see fig. 2 of Kamanzi et al. [10].). This could arise because only partial replacement of the acetate buffer by phosphate buffer is required to shift the acid‐base equilibrium, whereas ionic strength depends on the concentrations of all ions and therefore approaches its final value only after more extensive buffer exchange. As the instability driving fusion resulting from conversion of the cationic form of the ionizable lipid to the neutral form may be suggested to be most pronounced for the small vesicles as opposed to the larger LNP containing mRNA (see fig. 3 of Kulkarni et al. [20].), this may result in preferential intra‐vesicular fusion as compared with fusion involving the LNP containing mRNA. In the case of the two‐step process the ionic strength in combination with EtOH is clearly the fusion driver as the pH does not change. Note that the size of the LNP mRNA systems decreases as these systems are dialyzed against PBS (see Figure 1B and Figure S6), consistent with conversion of the ionizable lipid from the cationic, bilayer‐forming state to the neutral oil form but inconsistent with any further fusion.

**FIGURE 6:**
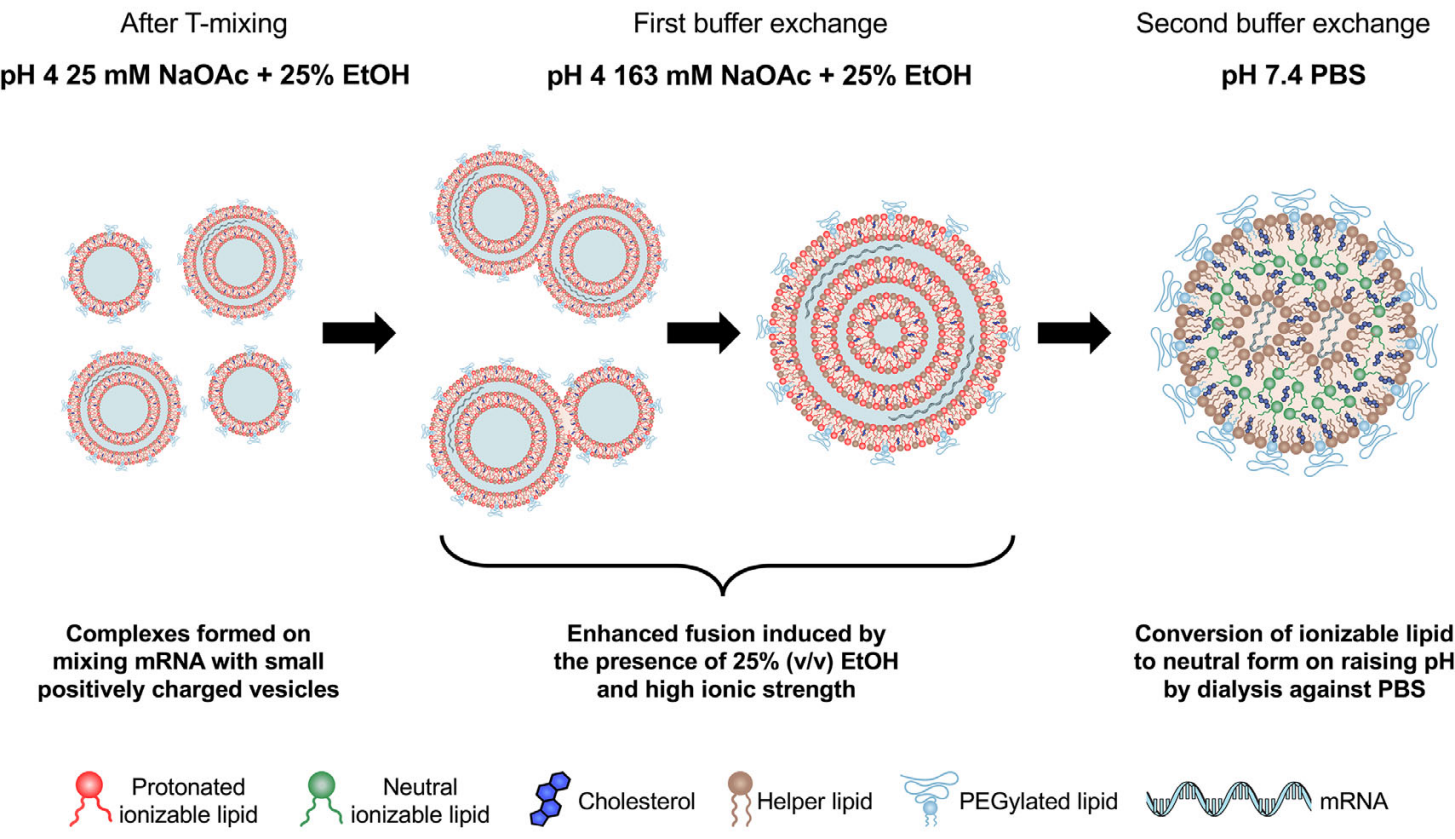
Proposed mechanism of the two‐step buffer exchange process. After T‐mixing in acidic conditions, complexes are formed from small positively charged vesicles and mRNA. Here they are drawn as multilayer systems where the mRNA is intercalated between apposed bilayers, see Radler et al. [21]. During the first buffer exchange against pH 4 163 mM NaOAc + 25% EtOH, fusion occurs between these complexes, resulting in the formation of larger complexes. In the second buffer exchange against pH 7.4 PBS, the EtOH is removed, and the ionizable lipid is neutralized, resulting in LNP mRNA systems consisting of an external monolayer of the bilayer‐forming lipids PC and cholesterol, the mRNA in polar regions surrounded by bilayer‐forming lipids, and the ionizable lipids in a central oil droplet.

Finally, with regard to the observation that LNP mRNA systems containing elevated levels of mRNA are more transfection potent, it is clear from Figure 3D,E that these systems result in enhanced intracellular delivery of mRNA. LNP mRNA systems that contain elevated levels of mRNA are larger and contain more ionizable lipid per LNP. Given the pivotal role of ionizable lipid in facilitating endosomal escape, this would be expected to enhance mRNA delivery to the cytoplasm.

## 4 Conclusion

LNP mRNA systems prepared by encouraging fusion at pH 4 due to the presence of 25% (v/v) EtOH exhibit improved transfection potency in vitro and in vivo as compared to LNP mRNA systems that only undergo dialysis against PBS. The results also indicate that upon i.v. administration, the eLNP mRNA systems exhibit enhanced transfection in the liver and spleen as compared to the cLNP mRNA formulations. Higher spleen transfection may arise due to eLNP having a larger particle size as larger particles are more readily accumulated by the spleen [22, 23]. As there has been limited investigation in studies relating LNP mRNA copy number to transfection potency, despite its clear clinical relevance, we propose an alternative post‐mixing protocol that yields higher mRNA loading with improved transfection properties and can be integrated into many existing formulation methods.

## 5 Methods and Materials

### 5.1 Materials

The ionizable lipids norMC3 and NTX‐001 were kindly provided by Dr. Marco Ciufolini. The lipids 1,2‐distearoylphosphatidylcholine (DSPC), 1‐palmitoyl‐2‐oleoylphosphatidylcholine (POPC), and 1,2‐dimyristoyl‐rac‐glycero‐3‐methoxypolyethylene glycol‐2000 (PEG‐DMG) were purchased from Avanti Polar Lipids, while cholesterol was obtained from Sigma‐Aldrich. The lipophilic dye, 3,3′‐dioctadecyloxacarbocyanine (DiO), was obtained from Thermo Fisher Scientific. Sodium acetate, sodium citrate tribasic dihydrate, tannic acid, and gold (III) chloride trihydrate were obtained from Sigma‐Aldrich. Firefly luciferase (FLuc) mRNA and Cy5‐FLuc mRNA were kindly provided by Dr. Andrew J. Varley (RNA Core at UBC). FLuc used in the SI in vitro transfection experiments was obtained from Hongene Biotech. Dulbecco's modified Eagle medium (DMEM), fetal bovine serum (FBS), and the cell surface marker CD62P‐PE were purchased from Thermo Fisher Scientific.

### 5.2 LNP Formulation

Lipid components (norMC3 or NTX‐001/DSPC or POPC/cholesterol/PEG‐DMG) were dissolved in EtOH to a final concentration of 5–10 mM total lipids and at a ratio of 50/10/38.5/1.5 mol%, respectively. The ethanolic lipid solution was then rapidly mixed with an aqueous buffer composed of pH 4 25 mM sodium acetate (unless otherwise noted) containing mRNA (FLuc, Cy5‐FLuc, or NanoLuc) at an amine‐to‐phosphate ratio (*N/P*) of 6. Rapid mixing was performed using a T‐junction mixer at a ratio of 1:3 EtOH/aqueous phase (v/v) with a total flow rate of 20 mL/min. All dialysis steps were performed using Spectra/Por dialysis membranes (MWCO: 12–14 kD; diameter: 16 mm). For one‐step buffer exchange, the resulting pH 4 dispersion was dialyzed overnight against 500–1000‐fold volume of pH 7.4 PBS. In the two‐step buffer exchange procedure, pH 4 vesicles were first dialyzed against 500–1000‐fold volume of pH 4 163 mM sodium acetate buffer containing varying amounts of EtOH (0%, 15%, 25%, or 35%) for 6 h prior to overnight dialysis against 500–1000‐fold volume of pH 7.4 PBS. In both instances, the resulting LNPs were then sterile filtered with a 0.2 µm Supor membrane syringe filter (Pall Corporation) and subsequently concentrated using 10 KDa Amicon ultracentrifugation units (EMD Millipore Corporation) prior to further characterization. In empty LNP formulations, mRNA was not added to the aqueous buffer while all other procedures described above remained the same. In LNP formulations used for CLiC analysis, 1 mol% DiO was incorporated and Cy5‐mRNA encapsulated. In LNP‐GNP formulations, 5 nm negatively charged GNPs were added (2.2E + 13 GNP/µmol lipid) to the aqueous buffer instead of mRNA while all other procedures described above remained the same.

### 5.3 LNP Lipid and RNA Quantification

Total lipid concentration was determined using the Cholesterol E Kit (Wako Diagnostics). The mRNA encapsulation efficiency and concentration were quantified using the Quant‐iT RiboGreen RNA Assay Kit (Invitrogen). *N/P* values are derived from total lipid and mRNA concentrations. Both absorption (lipid quantification) and fluorescence (RNA quantification) were measured using a Spark Multimode Microplate reader (Tecan).

### 5.4 LNP Size and Particle Concentration Analysis

Particle size analysis was determined through dynamic light scattering using a Zetasizer Nano (Malvern Instruments Inc.). Particle concentration was determined using a Zetasizer Advance Ultra. For LNP‐GNP formulations, cryo‐TEM micrographs were used to manually measure the diameters of 200 particles per LNP formulation via the software Fiji (ImageJ), and to also tabulate the number of GNPs per LNP.

### 5.5 Cryogenic Transmission Electron Microscopy (Cryo‐TEM)

Cryo‐TEM imaging was performed at the UBC High Resolution Macromolecular Cryo‐Electron Microscopy Facility (HRMEM) or at the UBC Bioimaging Facility. LNPs were concentrated to a lipid concentration of 15–20 mg/mL. The resulting dispersion was then added (3 µL) to glow‐discharged copper grids and plunge‐frozen in liquid ethane with an FEI Mark IV Vitrobot to generate vitreous ice. Grids were stored in liquid nitrogen prior to imaging. An FEI LaB6 G2 TEM operating at 200 kV under low‐dose conditions equipped with an FEI Eagle 4K CCD camera was used to image all samples unless otherwise specified. A magnification of 55 000x with a nominal under focus of 1–2 µm was used for sample imaging. Cryo‐TEM micrographs in Figure S9 were obtained from an FEI Tecnai G2 Twin TEM operating at 200 kV low‐dose conditions with a Gatan cryo sample holder. An FEI Eagle 4K‐bottom mount CCD camera was used for imaging at 29 000x and 50 000x magnification levels.

### 5.6 CLiC Flow‐Cell Preparation

CLiC imaging was performed using a flow‐cell designed similarly to those previously reported [11, 12]. Briefly, the flow‐cell consisted of two glass coverslips (25 × 25 mm, 200 µm thick) separated by a 30 µm double‐sided adhesive spacer. The bottom coverslip contained an array of cylindrical microwells (0.5 µm in height, 3 µm in diameter) used to confine individual freely diffusing particles within the focal plane of the imaging microscope.

To minimize particle interaction with glass surfaces, both coverslips were functionalized with 5 kDa polyethylene (PEG) brushes using a silane‐based cloud‐point PEGylation procedure as described previously [12]. The assembled flow‐cells were then mounted in a custom microfluidic chuck to interface with the CLiC pusher lens during experiments.

### 5.7 Single‐Particle CLiC‐ALEX Microscopy

Single‐particle imaging was carried out using CLiC‐ALEX microscopy, as previously reported (see Kamanzi et al. [12] for details). Briefly, the setup comprised of a Nikon Ti‐E inverted microscope equipment with 488 and 647 nm excitation lasers, an acousto‐optic tunable filter (AOTF) for rapid laser switching, a function generator for synchronized triggering, and two Andor iXon Ultra897 EMCCD cameras. The excitation beams were co‐aligned and directed through a 100X TIRF oil‐immersion objective (NA 1.49). The CLiC pusher lens was actuated by a piezoelectric nanopositioner to bring the flow‐cell surfaces into contact, thereby trapping freely diffusing particles within microwells.

Fluorescence emission was collected through the same objective and relayed to the two cameras using a dual‐band dichroic mirror and emission filter (Chroma TRF59906, 488/640 nm). A long‐pass filter (Chroma AT610lp) was then used to split emission signals into green and red detection channels corresponding to DiO and Cy5 fluorescence.

### 5.8 CLiC‐Alex Data Acquisition and Analysis

During experiments, camera exposure times were synchronized with the AOTF to enable both alternating laser excitation (ALEX) and simultaneous excitation modes. This approach allowed for improved quantification of DiO and Cy5 direct excitation by correcting for both bleed‐through and dye‐dye interaction through Förster resonance energy transfer (FRET).

Single‐particle trajectories were extracted from wells containing a single diffusing particle and used to compute mean‐square‐displacement (MSD) curves for hydrodynamic radius estimation. Fluorescence intensities of individual particles were quantified across all channels. Cross‐channel colocalization of DiO and Cy5 signals was then used to classify individual particles as empty or loaded. Finally, mRNA copy number per loaded particle was estimated by normalizing Cy5 intensities of the particles to the average Cy5 signal of individual mRNA molecules.

### 5.9 GNP Synthesis

Five‐nanometer gold nanoparticles (GNPs) were prepared by reduction of gold chloride salt. A gold salt solution was prepared by dissolving 58 mg HAuCl_4_·3H_2_O in 400 mL deionized (DI) H_2_O. A reduction solution was prepared separately by dissolving 200 mg Na_3_Citrate·2H_2_O, 50 mg tannic acid, and 17.5 mg K_2_CO_3_ in 100 mL DI‐H_2_O. Both solutions were heated separately to 60°C on stirrers. At 60°C, the reduction solution was added all at once to the gold salt solution while being stirred at 1150 rpm. A red color formed immediately, and the mixture was heated to 95°C and then placed in cold ice water to cool down. GNPs were purified and concentrated using 10 kDa Amicon ultracentrifugation units (EMD Millipore Corporation).

### 5.10 Cell Culture, Transfection, and Viability Studies

The human hepatocarcinoma cell line, Huh7, was used for all in vitro experiments. Cells were cultured and maintained in DMEM media supplemented with 10% fetal bovine serum (FBS) at 37°C with 5% CO_2_. Cells were seeded in 96‐well microplates (10 000 cells/well) 24 h prior to transfection. LNP encapsulating FLuc mRNA were then added to cells at 0.003–3.0 µg/mL mRNA, and cells were allowed to incubate for another 24 h. After incubation, the media was removed, and 100 µL of Glo Lysis buffer (Promega) was added to lyse cells. Finally, the lysates were mixed at a 1:1 (v/v) ratio with luciferase assay substrate (Promega) in a 96‐well plate, and luminescence was quantified using a Spark Multimode Microplate reader (Tecan) with an integration time of 1000 ms. Absolute luminescence measurements were used in Figure 3, while luminescence normalized by total protein content (determined from BCA Assay) was used for Figures S10 and S12. In vitro cell viability studies were conducted using the LDH‐Glo Cytotoxicity Assay kit with Huh7 cells after 24 h incubation with LNP mRNA formulations.

### 5.11 Cell Uptake Studies

Huh7 cells were seeded in six‐well microplates (250 000 cells/well) 24 h prior to LNP mRNA treatment. LNPs encapsulating Cy5‐FLuc mRNA were then added to cells at a dose of 0.3 µg/mL mRNA and allowed to incubate for 24 h. After incubation, the media was removed, and the cells were trypsinized, treated with 1X PBS + 2% FBS, and washed twice with FACS buffer (1X PBS, 2% FBS, 1 mM ethylenediaminetetraacetic acid (EDTA), 0.1% sodium azide) before a final resuspension in 500 µL FACS buffer. 20 000 events gated on the viable cell population based on forward and side scatter properties (Figure S18) were collected on the BD CytoFLEX LX, where the median fluorescence intensity of Cy5 was used to assess the mRNA content in cells. The data was then analyzed using FlowJo (v10.10).

### 5.12 Ex Vivo Human Donor Platelet Transfection Using LNP mRNA

Human donor platelets were treated as previously described [16]. Briefly, donor platelets were mixed with 12 µg/mL LNP mRNA (NTX‐001/POPC/Cholesterol/PEG‐DMG at 50/10/38.5/1.5 mol%) and platelet additive solution (PAS) to a final concentration of 250 × 10^6^ platelets/mL and a PAS to plasma ratio (PAS:PPP) of 70:30. Platelets were then incubated at 37°C, where at 4 and 24 h post‐treatment, 40 × 10^6^ platelets were removed and pelleted before lysing in a total volume of 200 µL Glo‐lysis buffer (Promega). 50 µL of lysate was mixed at a 1:1 ratio with NanoLuc luciferase reagent (Promega), and luminescence was then recorded on a Spark Multimode Microplate reader (Tecan) with an integration time of 1000 ms. Platelet activation was measured with flow cytometry using the cell surface marker P‐selectin (CD62P) conjugated to the fluorophore phycoerythrin (PE).

### 5.13 In Vivo Bioluminescence Imaging of LNP mRNA Luciferase Transfection

Animal protocols used were approved by the Canadian Animal Care Committee at the University of British Columbia (UBC; protocol number A22‐0044). All animal work was conducted at the modified barrier animal facility at UBC. CD1 female mice (6–8 weeks old) were purchased from Charles River Laboratories and were maintained on a regular 12 h light/12 h dark cycle. Mice were individually weighed and imaged prior to intravenous (i.v.) and intramuscular (i.m.) administration of LNP FLuc mRNA (0.2 mg/kg and 0.1 mg/kg mRNA, respectively). IVIS bioluminescence imaging was performed at 4 and 24 h post‐injection. D‐luciferin substrate (Promega) was injected intraperitoneally (150 mg/kg) 10 min prior to imaging. Mice within the same dosing groups (*n* = 3) were then anesthetized with 3% isoflurane and placed in a nose cone supplying 3% isoflurane. A Xenogen IVIS Lumina II In Vivo Imaging System (PerkinElmer) was used for all experiments. The exposure time was set to 10 s for i.v. experiments and 30 s for i.m. experiments. Bioluminescence was determined by measuring the radiance (photons/s/cm^2^/steradian) of identified regions of interest using the Living Image software.

Immediately after the final imaging time point (24 h), mice were euthanized, and organs of interest were harvested. For i.v. experiments, the heart, lung, liver, spleen, and kidney were imaged ex vivo. For i.m. experiments, the liver, spleen, muscle, and inguinal lymph node were imaged ex vivo. All ex vivo imaging was performed with a 30 s exposure time, and bioluminescence was determined by quantifying the radiance (photons/s/cm^2^/steradian) for each excised organ using the Living Image software.

### 5.14 Organ Homogenization and Biodistribution Quantification

All excised organs were collected in Fastprep tubes 24 h post injection and immediately following ex vivo IVIS imaging. In each tube, 1 mL of 1X reporter lysis buffer (Promega) and a ¼″ ceramic bead was added prior to homogenization with a FastPrep‐24 Classic bead beating grinder and lysis system (MP). Organs were homogenized for two rounds at 4 m/s for 20s followed by centrifugation at 13 523 g for 10 min at 4°C. The resulting lysate was then collected and mixed at a 1:1 ratio with luciferase assay substrate (Promega) before adding to a 96‐well plate. Luminescence was quantified using a Spark Multimode Microplate reader (Tecan) and normalized against total protein.

### 5.15 BCA Assay

A Pierce BCA Protein Assay Kit (Thermo Fisher Scientific) was used to quantify total protein in organ and cell lysates. A standard curve containing bovine serum albumin was prepared in PBS starting at 2 mg/mL and serially diluted. In a 96‐well plate, 10 µL of standard and diluted organ or cell lysates were added into each well, followed by 190 µL of BCA reagent. The plate was incubated for 45 min at 37°C, and absorbance was then measured at 562 nm with a Spark Multimode Microplate reader (Tecan). The protein concentration of organ lysates was interpolated using the standard curve.

### 5.16 Statistical Analysis

All data were reported as the mean ± standard deviation or mean ± standard error of the mean. Comparisons between groups were analyzed by a one‐way analysis of variance (ANOVA) followed by Tukey's multiple comparisons test, two‐tailed unpaired *t*‐test, or Mann‐Whitney U test. Statistical significance was defined as: ns = nonsignificant, *p < 0.05, **p < 0.01, ***p < 0.001, ****p < 0.0001, with a 95% confidence interval. All statistical analyses were performed using GraphPad Prism 10.

## Author Contributions

**Yao Zhang**: conceptualization, investigation, writing – original draft, writing – review and editing, visualization, data curation, methodology, formal analysis, validation. **Jerry Leung**: investigation, methodology, validation, visualization, writing – review and editing, formal analysis, data curation. **Judy Cheng**: investigation, validation, methodology. **Arash Momeni**: investigation, methodology, writing – review and editing. **Albert Kamanzi**: investigation, conceptualization, writing – review and editing, data curation, formal analysis. **Ariadne Tuckmantel Bido**: investigation, validation. **Suiyang Liao**: validation, investigation, writing – review and editing. **Emma Kang**: investigation, validation, writing – review and editing. **Colton Strong**: investigation, validation, writing – review and editing. **Christian J. Kastrup**: funding acquisition, writing – review and editing, supervision, resources. **Sabrina Leslie**: funding acquisition, writing – review and editing, supervision, resources. **Miffy Hok Yan Cheng**: conceptualization, investigation, writing – review and editing, writing – original draft, validation, methodology, supervision. **Pieter R. Cullis**: conceptualization, investigation, writing – review and editing, writing – original draft, validation, methodology, supervision, funding acquisition, resources.

## Conflicts of Interest

A.K. and S.L. have financial interests in ScopeSys Inc. C.J.K. and P.R.C. have financial interests in companies developing RNA therapies, including NanoVation Therapeutics, SeraGene Therapeutics, Syrina Therapeutics, and Acuitas Therapeutics.

## Supporting information




**Supporting File**: adma75174‐sup‐0001‐SuppMat.pdf.

## Data Availability

The data that support the findings of this study are available from the corresponding author upon reasonable request.
