# Supplementary material for: Increasing Lipid Nanoparticle mRNA Copy Number Improves Transfection Potency
Source: Adv Mater. 2026 Sep 27;38(63):e75174. doi: 10.1002/adma.75174 (PMC13755665; doi:10.1002/adma.75174)
Supplement: Supplementary file 1 [file ADMA-38-e75174-s001.pdf]

SUPPLEMENTARY INFORMATION

Increasing Lipid Nanoparticle mRNA Copy Number Improves Transfection Potency

Yao Zhang[1,2,3], Jerry Leung[1,3], Judy Cheng[4], Arash Momeni[1], Albert Kamanzi[3], Ariadne Tuckmantel Bido[3], Suiyang Liao[1,2,3], Emma Kang[1,3], Colton Strong[1,3], Christian J. Kastrup[1,3,6], Sabrina Leslie[3,5], Miffy Hok Yan Cheng[1,7*], Pieter R Cullis[1*]

[1]Department of Biochemistry and Molecular Biology, University of British Columbia, Vancouver, BC, Canada V6T 1Z3

[2]School of Biomedical Engineering, University of British Columbia, Vancouver, BC, Canada V6T 1Z4

[3]Michael Smith Laboratories, University of British Columbia, Vancouver, BC, Canada V6T 1Z4

[4]Department of Psychiatry, Djavad Mowafaghian Centre for Brain Health, University of British Columbia, Vancouver, BC, Canada V6T 1Z3

[5]Department of Physics and Astronomy, University of British Columbia, Vancouver, BC, Canada V6T 1Z6

[6]Versiti Blood Research Institute, Milwaukee, WI, United States, 53226

[7]Faculty of Pharmaceutical Sciences, University of British Columbia, Vancouver, BC, Canada, V6T 1Z3

**Co-corresponding author*

Miffy Hok Yan Cheng: miffy.cheng@ubc.ca

Pieter R Cullis: pieterc@mail.ubc.ca

Keywords: lipid nanoparticles, gene therapy, drug delivery, nanomedicine, mRNA loading

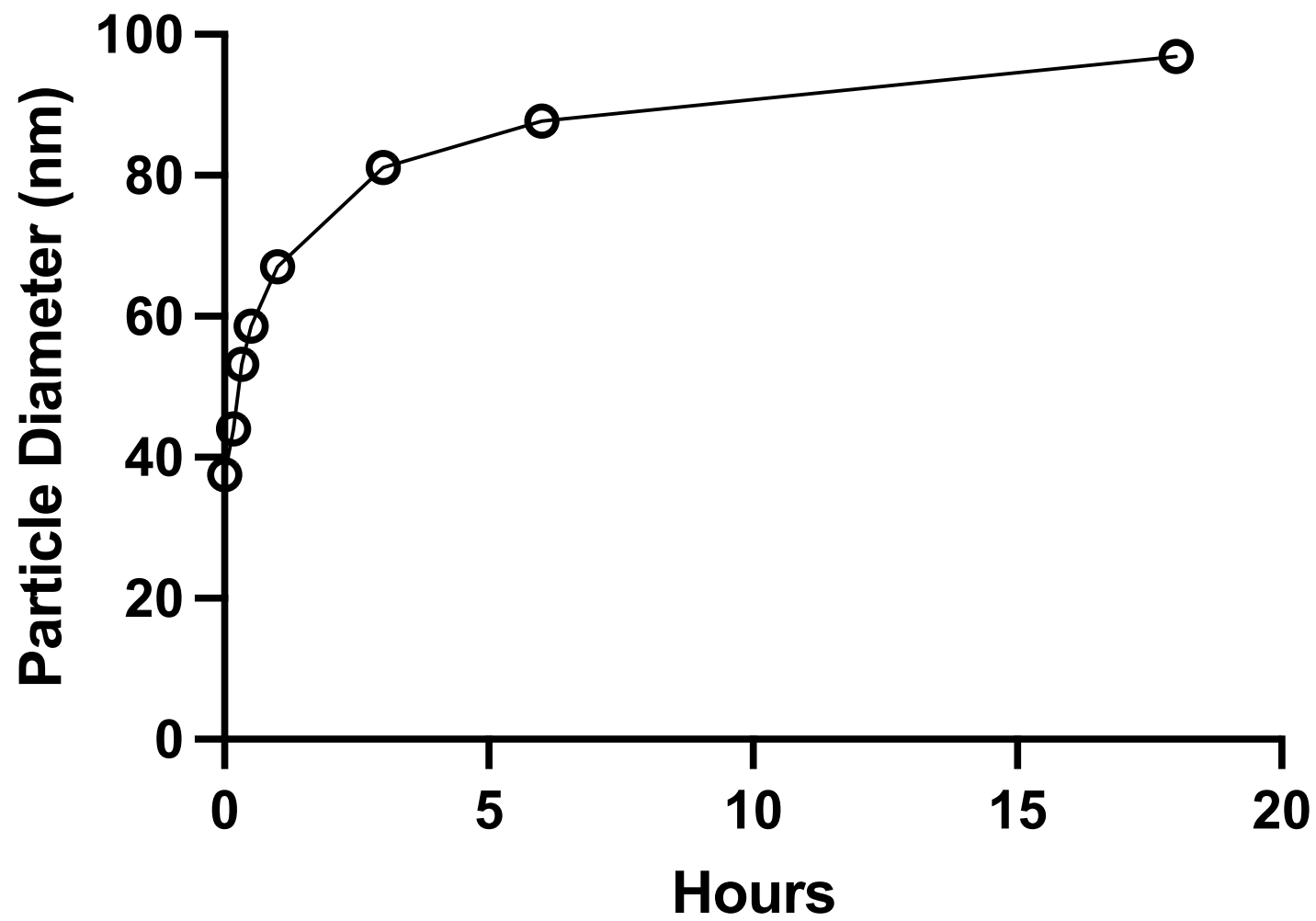

Figure S1. Fusion kinetics of LNP mRNA systems subjected to the fusogenic 163 mM NaOAc + 25% EtOH buffer. LNP mRNA (FLuc; N/P = 6) were formulated with an Onpattro-based composition consisting of norMC3/DSPC/Cholesterol/PEG-DMG (50/10/38.5/1.5 mol%, respectively) and were dialyzed against pH 4 163 mM NaOAc + 25% EtOH. The particle size (Z-average) as measured by DLS was performed after 0 min, 10 min, 20 min, 30 min, 1 h, 3 h, 6 h, and 18 h of dialysis.

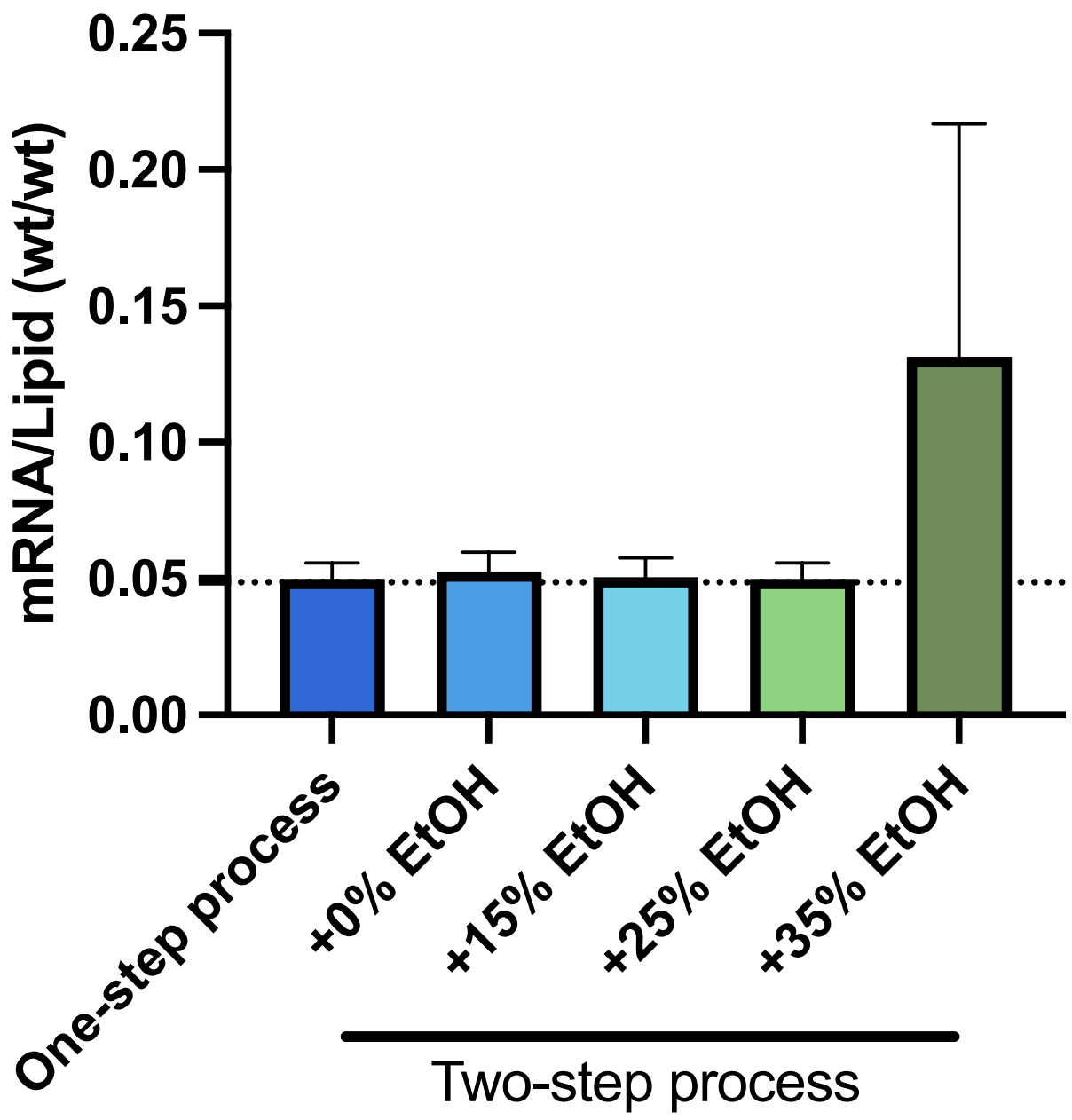

Figure S2. LNP mRNA formulations dialyzed in the presence of 35% EtOH results in loss of lipids. LNP mRNA (FLuc; N/P = 6) were formulated with an Onpattro-based composition consisting of norMC3/DSPC/Cholesterol/PEG-DMG (50/10/38.5/1.5 mol%, respectively) and were subjected to a one-step (PBS dialysis) or two-step (163 mM NaOAc + 0%/15%/25%/35% EtOH dialysis prior to PBS dialysis) buffer exchange. The mRNA concentration was determined through the RiboGreen assay and lipid concentration was measured with the Cholesterol E Kit. Dotted line represents the theoretical ratio of mRNA/lipid (wt/wt) = 0.049.

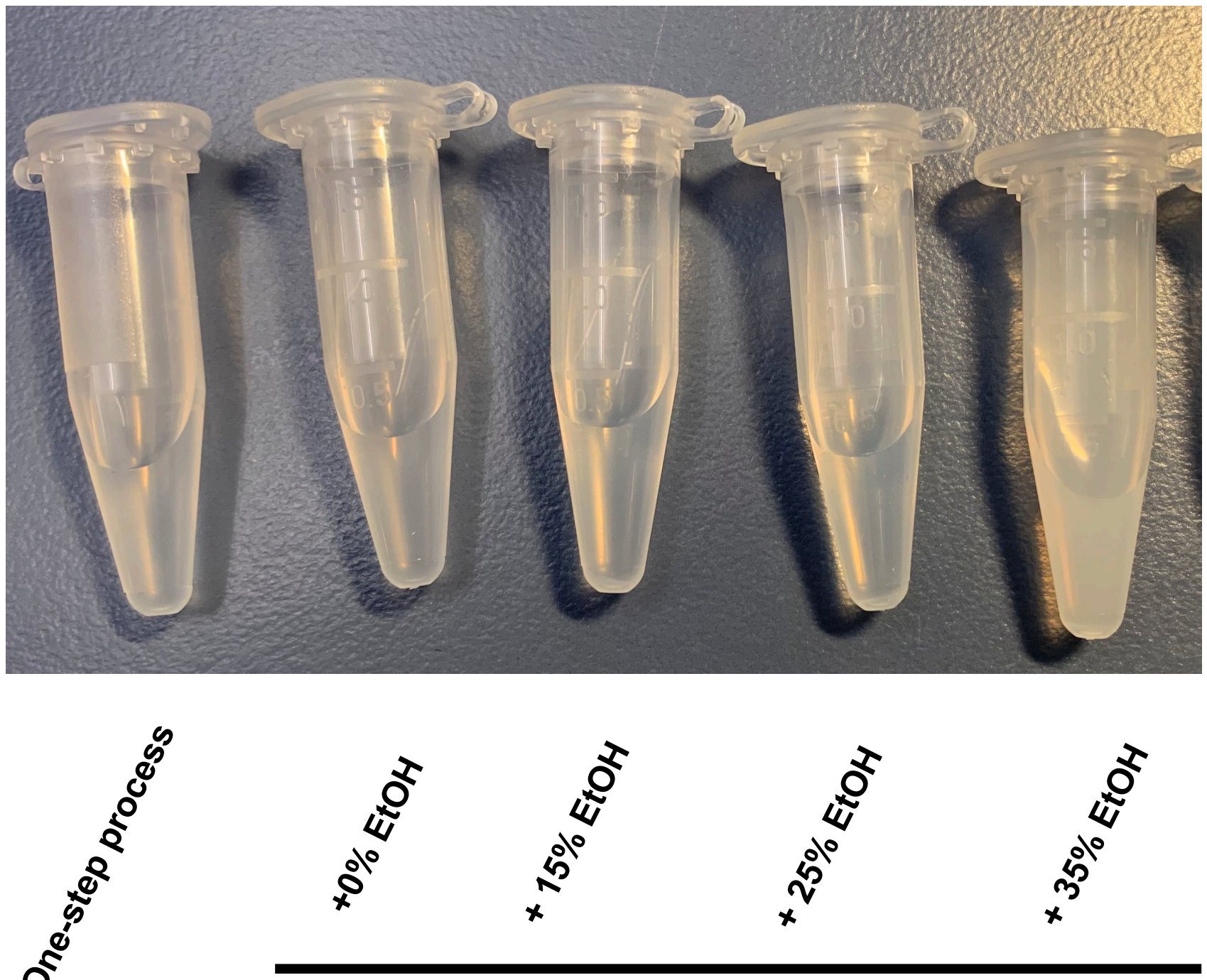

Figure S3. LNP mRNA formulations dialyzed in the presence of higher EtOH content appear cloudier upon visual inspection, indicating the presence of larger particles. LNP mRNA (FLuc; N/P = 6) were formulated with an Onpattro-based composition consisting of norMC3/DSPC/Cholesterol/PEG-DMG (50/10/38.5/1.5 mol%, respectively) and were subjected to a one-step (PBS dialysis) or two-step (163 mM NaOAc + 0%/15%/25%/35% EtOH dialysis prior to PBS dialysis) buffer exchange.

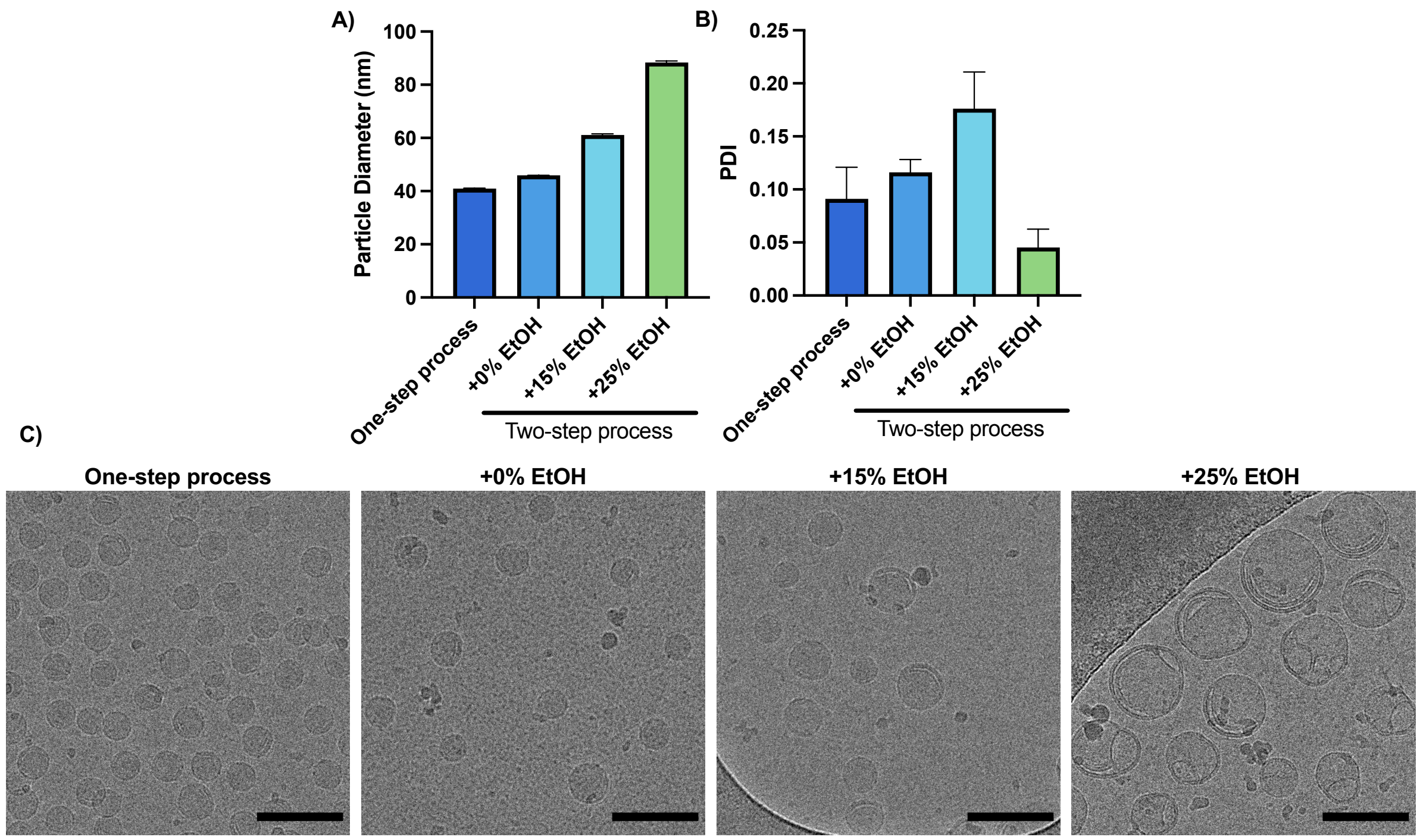

Figure S4. LNP-LNP fusion arising from the two-step buffer exchange process is dependent on the LNP and not on the cargo. Empty LNPs were formulated with an Onpattro-based composition consisting of norMC3/DSPC/Cholesterol/PEG-DMG (50/10/38.5/1.5 mol%, respectively) and were subjected to a one-step (pH 7.4 PBS dialysis) or two-step (pH 4 163 mM NaOAc + 0%/15%/25% EtOH dialysis prior to pH 7.4 PBS dialysis) buffer exchange. Empty LNPs suspended in pH 7.4 PBS were then characterized. **A)** Particle size (Z-average) and **B)** PDI measured by DLS are shown in bar graphs. **C)** Cryo-TEM micrographs of the empty LNP formulations. Scale bar = 100 nm.

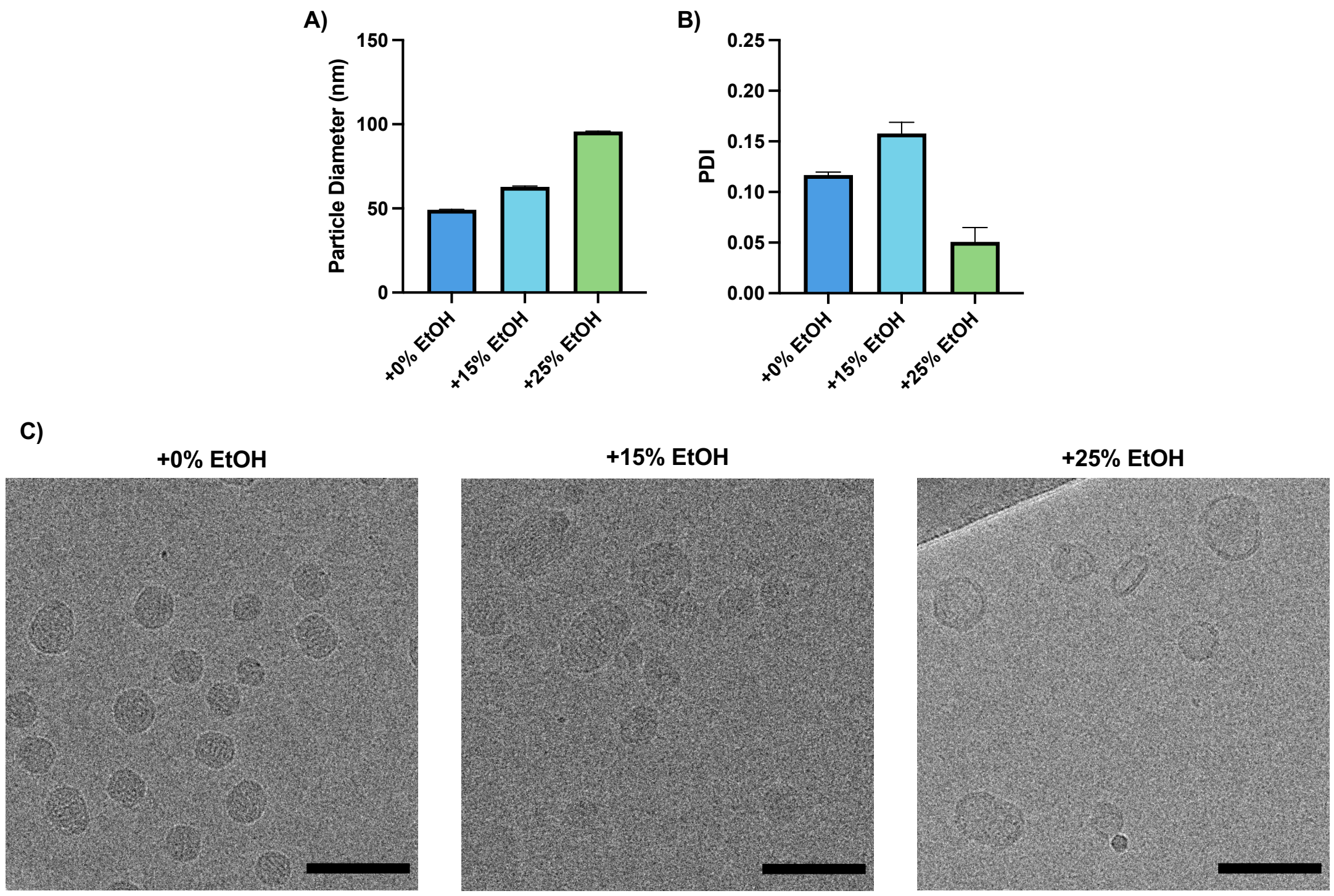

Figure S5. LNP-LNP (empties) fusion arising from the two-step buffer exchange occurs at pH 4. Empty LNPs were formulated with an Onpattro-based composition consisting of norMC3/DSPC/Cholesterol/PEG-DMG (50/10/38.5/1.5 mol%, respectively) and were dialyzed against pH 4 163 mM NaOAc + 0%/15%/25% EtOH prior to final dialysis against pH 4 163 mM NaOAc. Empty LNPs suspended in pH 4 163 mM NaOAc were then characterized. **A)** Particle size (Z-average) and **B)** PDI measured by DLS are shown in bar graphs. **C)** Cryo-TEM of pH 4 LNPs. Scale bar = 100 nm.

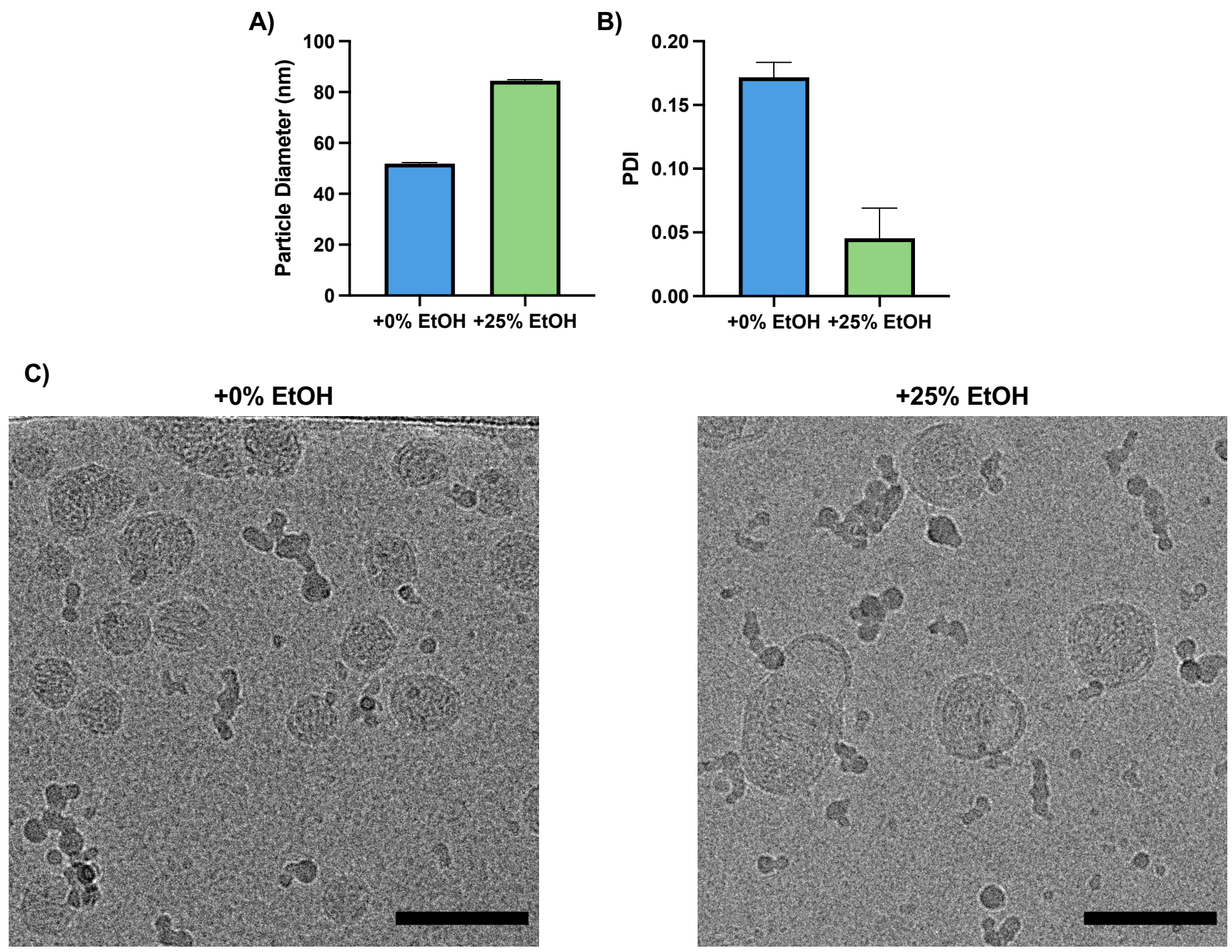

Figure S6. LNP-LNP (mRNA loaded) fusion arising from the two-step buffer exchange occurs at pH 4. LNP mRNA (FLuc; N/P = 6) were formulated with an Onpattro-based composition consisting of norMC3/DSPC/Cholesterol/PEG-DMG (50/10/38.5/1.5 mol%, respectively) and were dialyzed against pH 4 163 mM NaOAc + 0% or 25% EtOH prior to final dialysis against pH 4 163 mM NaOAc. LNP mRNA suspended in pH 4 163 mM NaOAc were then characterized. **A)** Particle size (Z-average) and **B)** PDI measured by DLS are shown in bar graphs. **C)** Cryo-TEM of pH 4 LNPs. Scale bar = 100 nm.

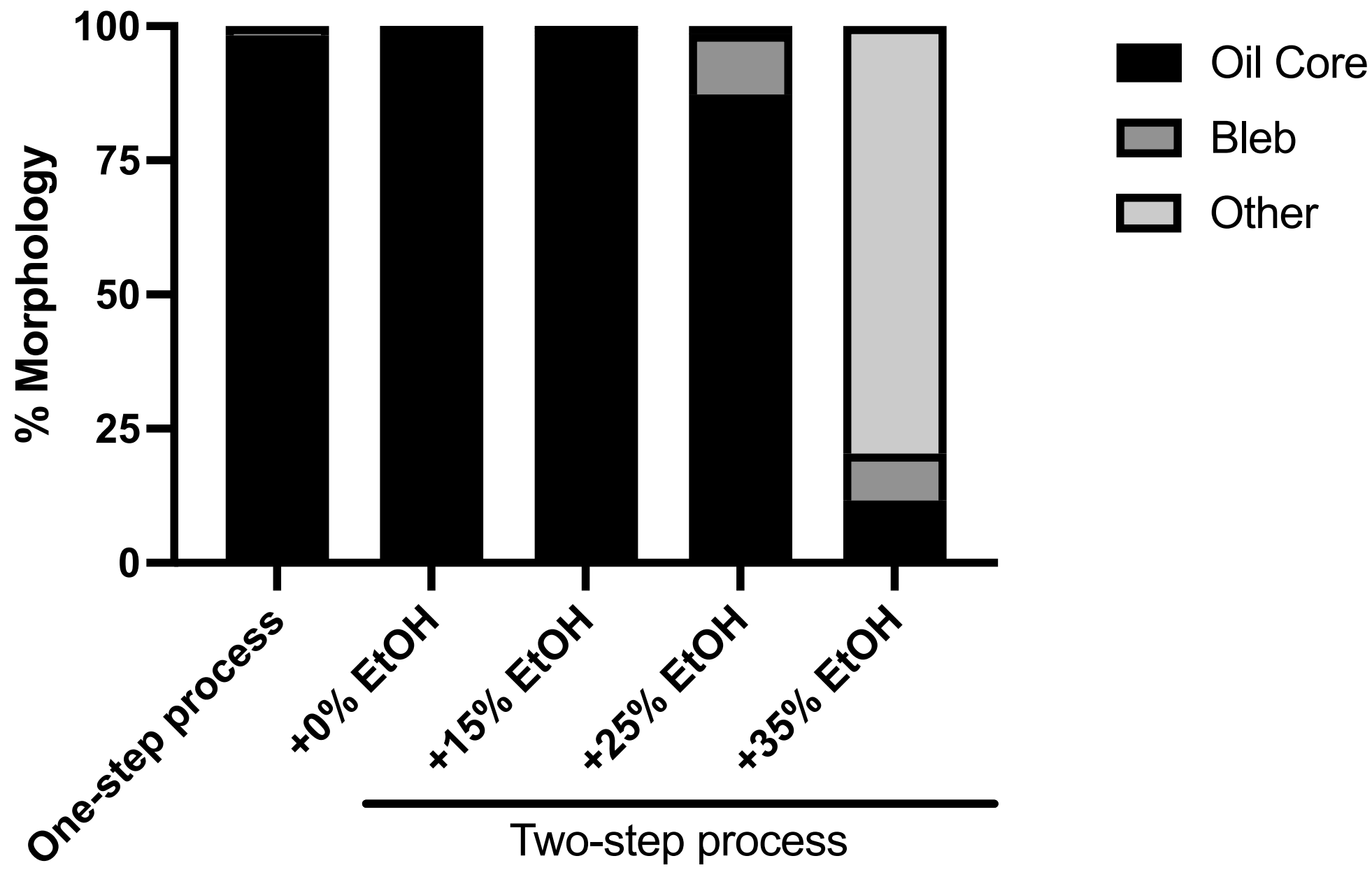

Figure S7. LNP mRNA formulations display a predominately oil core morphology except for particles dialyzed against 163 mM NaOAc + 35% EtOH. LNP mRNA (FLuc; N/P = 6) were formulated with an Onpattro-based composition consisting of norMC3/DSPC/Cholesterol/PEG-DMG (50/10/38.5/1.5 mol%, respectively) and were subjected to a one-step (PBS dialysis) or two-step (163 mM NaOAc + 0%/15%/25%/35% EtOH dialysis prior to PBS dialysis) buffer exchange. The LNP morphologies from cryo-TEM micrographs were then classified into three categories (Oil Core, Bleb, or Other). “Other” refers to particles that resemble a liposome structure, have distinct internal structure, or appear aggregated. Cryo-TEM morphology was manually classified (n = 300 particles).

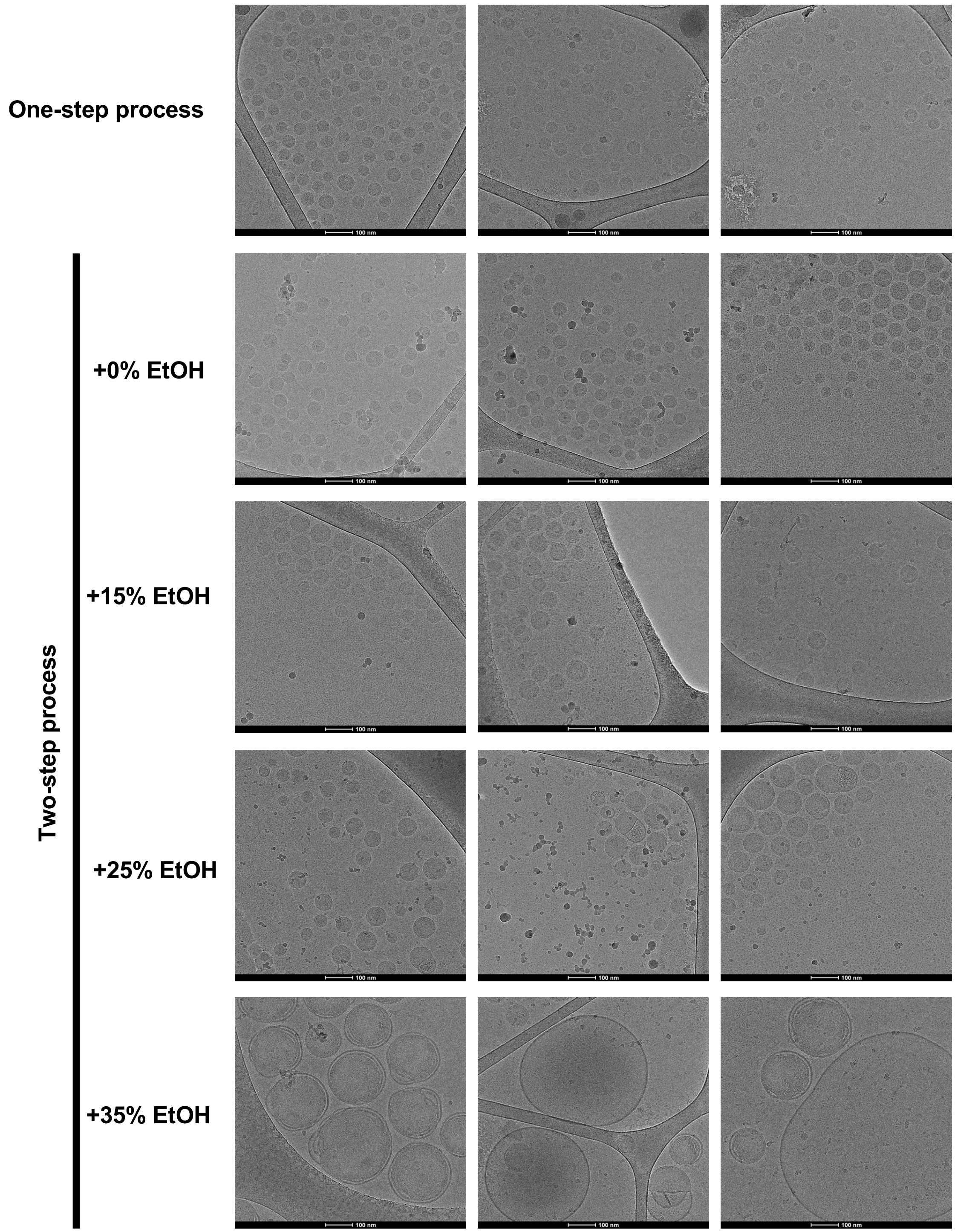

Figure S8. Additional representative cryo-TEM micrographs of Onpattro-based norMC3 LNP mRNA formulations. LNP mRNA (FLuc; N/P = 6) were formulated with an Onpattro-based composition consisting of norMC3/DSPC/Cholesterol/PEG-DMG (50/10/38.5/1.5 mol%, respectively) and were subjected to a one-step (PBS dialysis) or two-step (163 mM NaOAc + 0%/15%/25%/35% EtOH dialysis prior to PBS dialysis) buffer exchange. The dispersion was then concentrated and imaged.

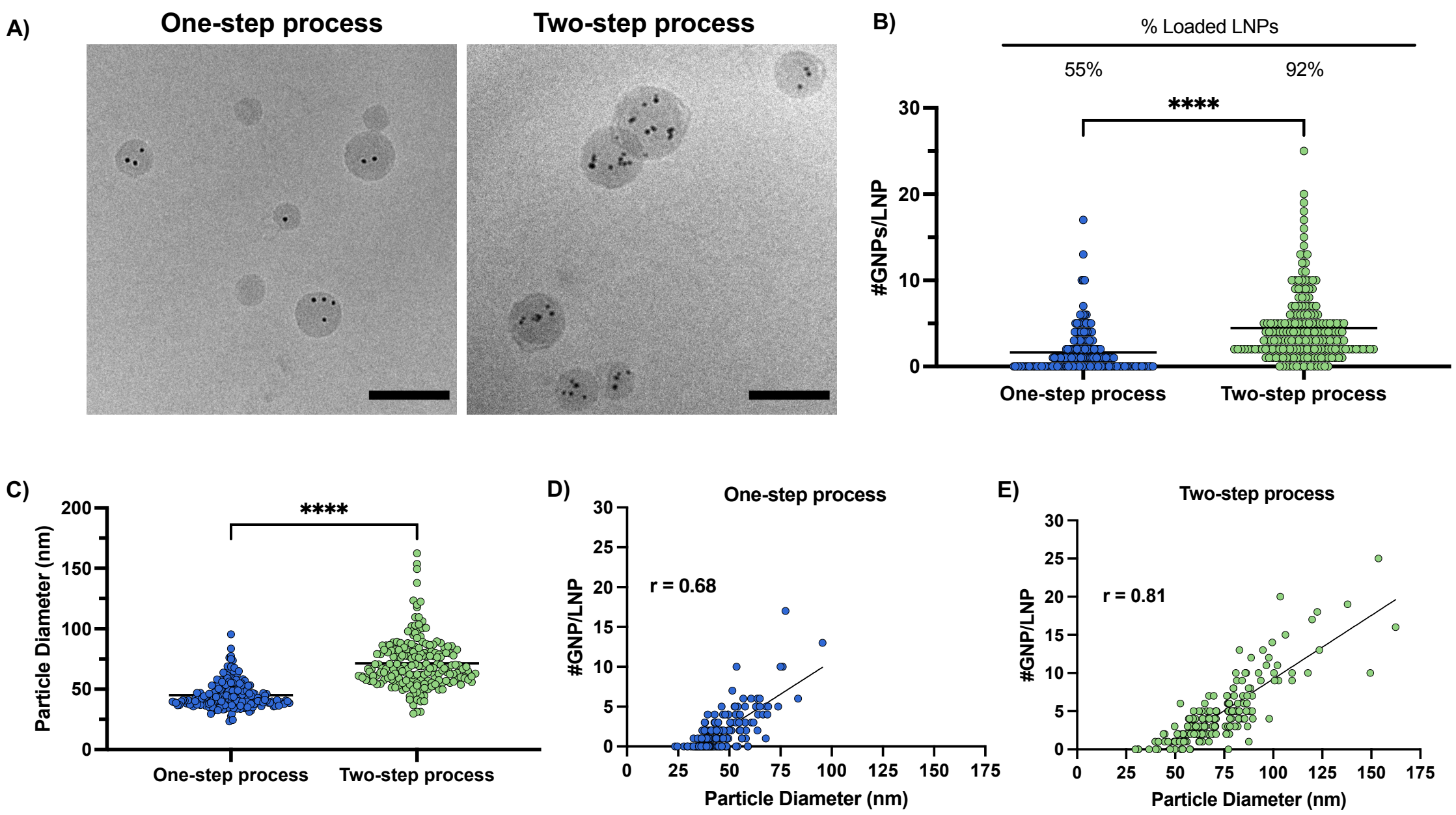

Figure S9. LNP GNP systems formulated using the two-step buffer exchange process yields an increased #GNP/LNP with a lower fraction of empty LNPs compared to the standard one-step buffer exchange procedure. LNP GNP (5 nm; 2.2E+13 GNP particles/μmol lipid) were formulated with a lipid composition consisting of norMC3/DSPC/Cholesterol/PEG-DMG (50/10/38.5/1.5 mol%, respectively) and were subjected to a one-step (PBS dialysis) or two-step (163 mM NaOAc + 25% EtOH dialysis prior to PBS dialysis) buffer exchange. **A)** Cryo-TEM micrographs of the different LNP GNP. Scale bar = 100 nm. **B)** Quantification of the number of GNP per LNP and percent empty LNP (mean, n = 200). Data were analyzed through a Mann-Whitney U test, ****p < 0.0001. **C)** Particle diameters of the LNP GNP manually measured from cryo-TEM micrographs using Fiji software (mean, n = 200). Data were analyzed through a Mann-Whitney U test, ****p < 0.0001. **D–E)** Correlation between the particle diameter and #GNP/LNP (Spearman r shown) for the (D) one-step buffer exchange process and (E) the two-step buffer exchange process.

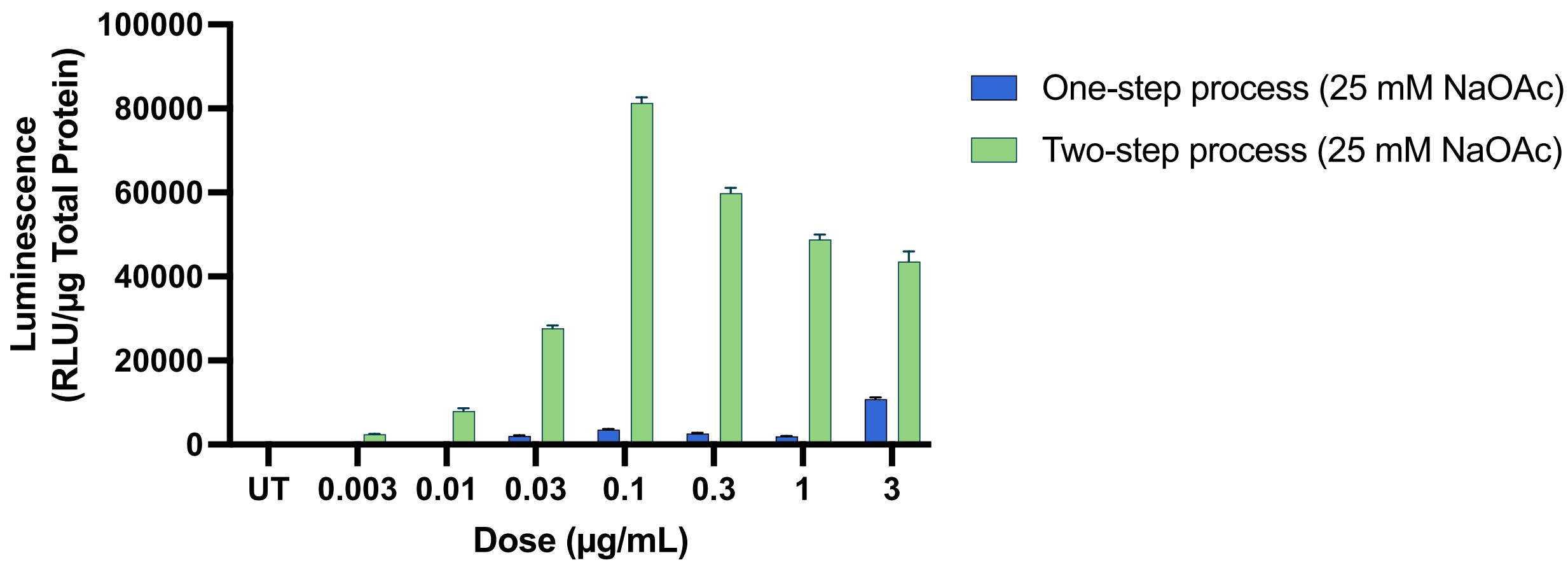

Figure S10. LNP mRNA formulated with the two-step buffer exchange process improves *in vitro* transfection across a range of doses compared to LNP mRNA produced from a one-step buffer exchange. LNP mRNA (FLuc; N/P = 6) were formulated with 25 mM NaOAc and a lipid composition consisting of norMC3/DSPC/Cholesterol/PEG-DMG (50/10/38.5/1.5 mol%, respectively). The dispersion was then subjected to a one-step (PBS dialysis) or two-step (163 mM NaOAc + 25% EtOH dialysis prior to PBS dialysis) buffer exchange. Luminescence (normalized to total protein) readout of Huh7 cells after 24 h incubation with LNP mRNA formulations (mean ± S.E.M., n = 3). UT=untreated cells.

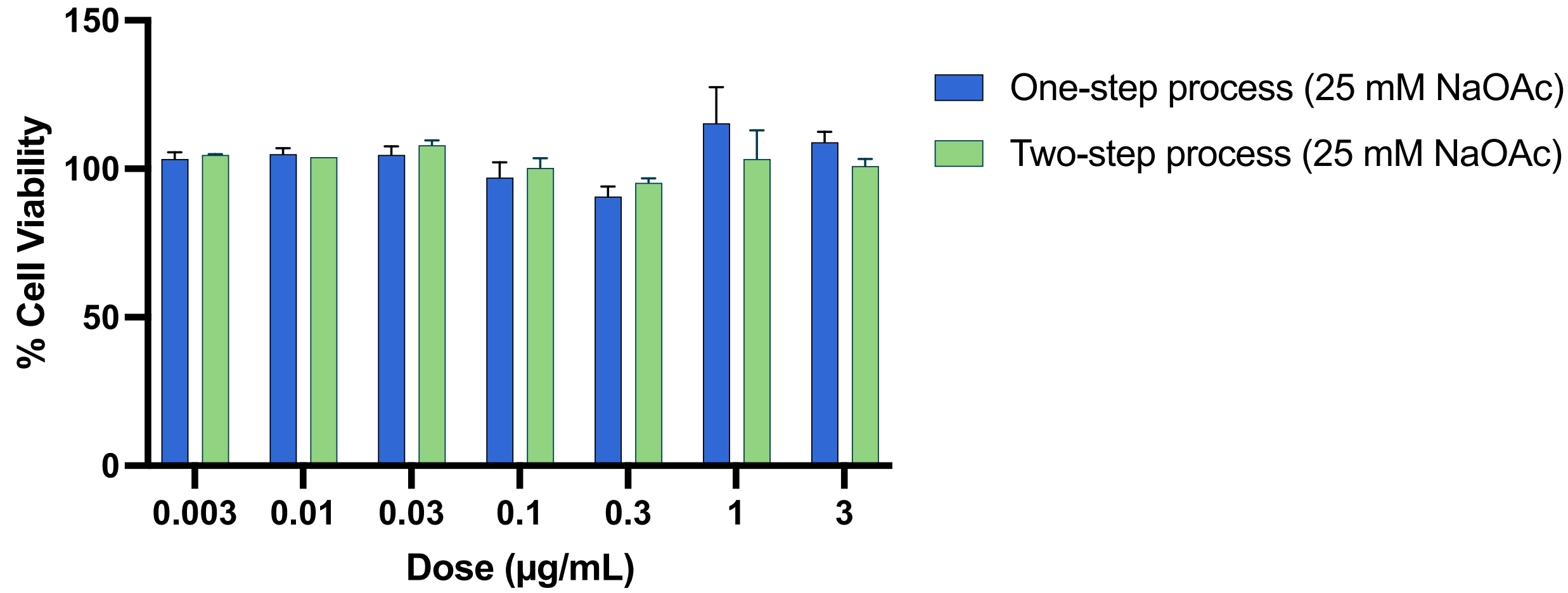

Figure S11. LNP mRNA formulated with the two-step buffer exchange process does not affect cell viability. LNP mRNA (FLuc; N/P = 6) were formulated with 25 mM NaOAc and a lipid composition consisting of norMC3/DSPC/Cholesterol/PEG-DMG (50/10/38.5/1.5 mol%, respectively). The dispersion was then subjected to a one-step (PBS dialysis) or two-step (163 mM NaOAc + 25% EtOH dialysis prior to PBS dialysis) buffer exchange. Cell viability was determined using the LDH-Glo^{TM} Cytotoxicity assay with Huh7 cells after 24 h incubation with LNP mRNA formulations (mean ± S.E.M., n = 3).

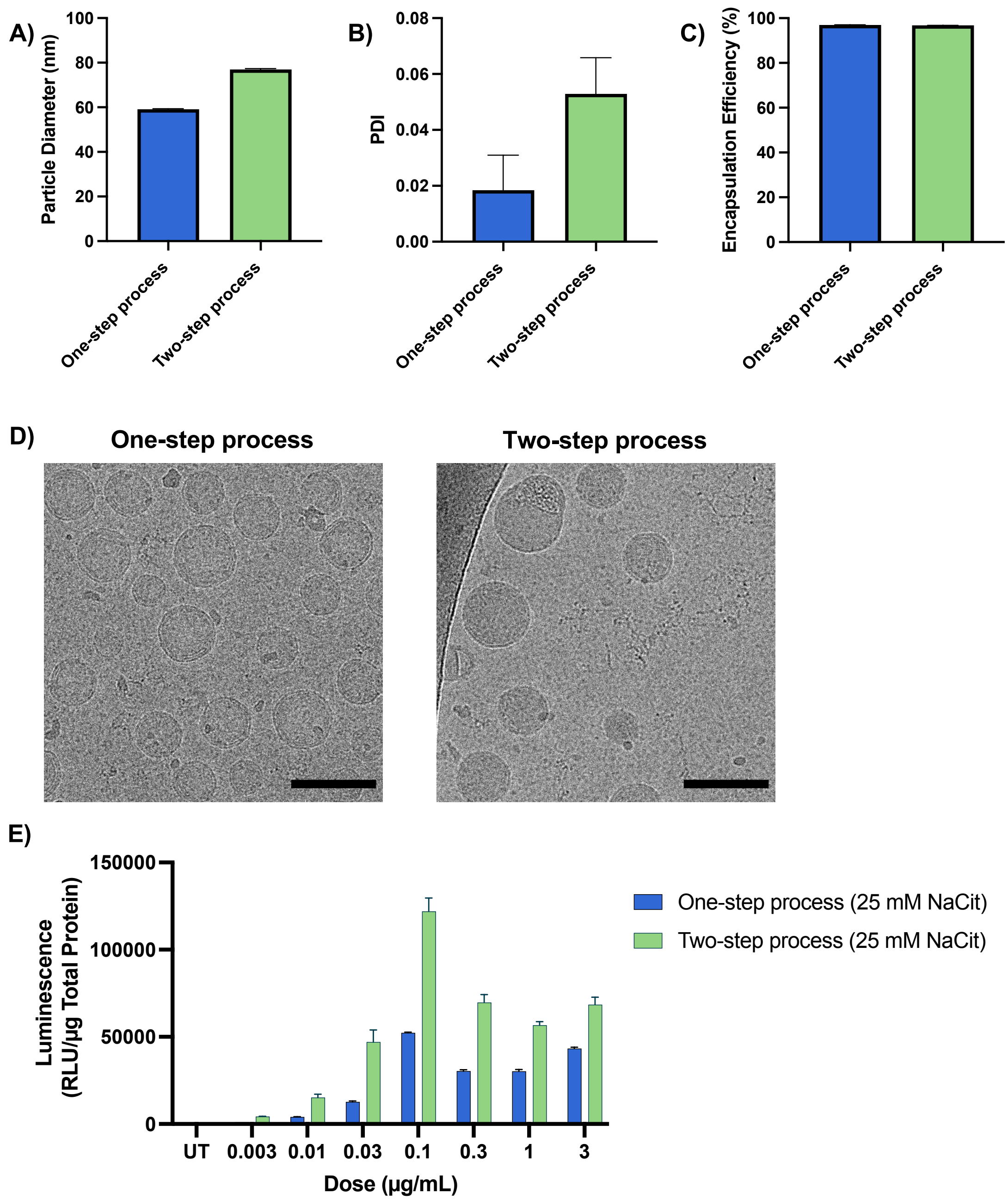

Figure S12. The two-step buffer exchange process can also be applied to LNP mRNA systems formulated with 25 mM sodium citrate, resulting in improved *in vitro* transfection potency compared to the one-step buffer exchange process. LNP mRNA (FLuc; N/P = 6) were formulated with 25 mM sodium citrate (NaCit) and a lipid composition consisting of norMC3/DSPC/Cholesterol/PEG-DMG (50/10/38.5/1.5 mol%, respectively). The dispersion was then subjected to a one-step (PBS dialysis) or two-step (163 mM NaOAc + 25% EtOH dialysis prior to PBS dialysis) buffer exchange. **A)** Particle size (Z-average), **B)** PDI, **C)** encapsulation efficiency, and **D)** cryo-TEM micrographs. Scale bar = 100 nm. **E)** Luminescence (normalized to total protein) readout of Huh7 cells after 24 h incubation with LNP mRNA formulations (mean ± S.E.M., n = 3). UT=untreated cells.

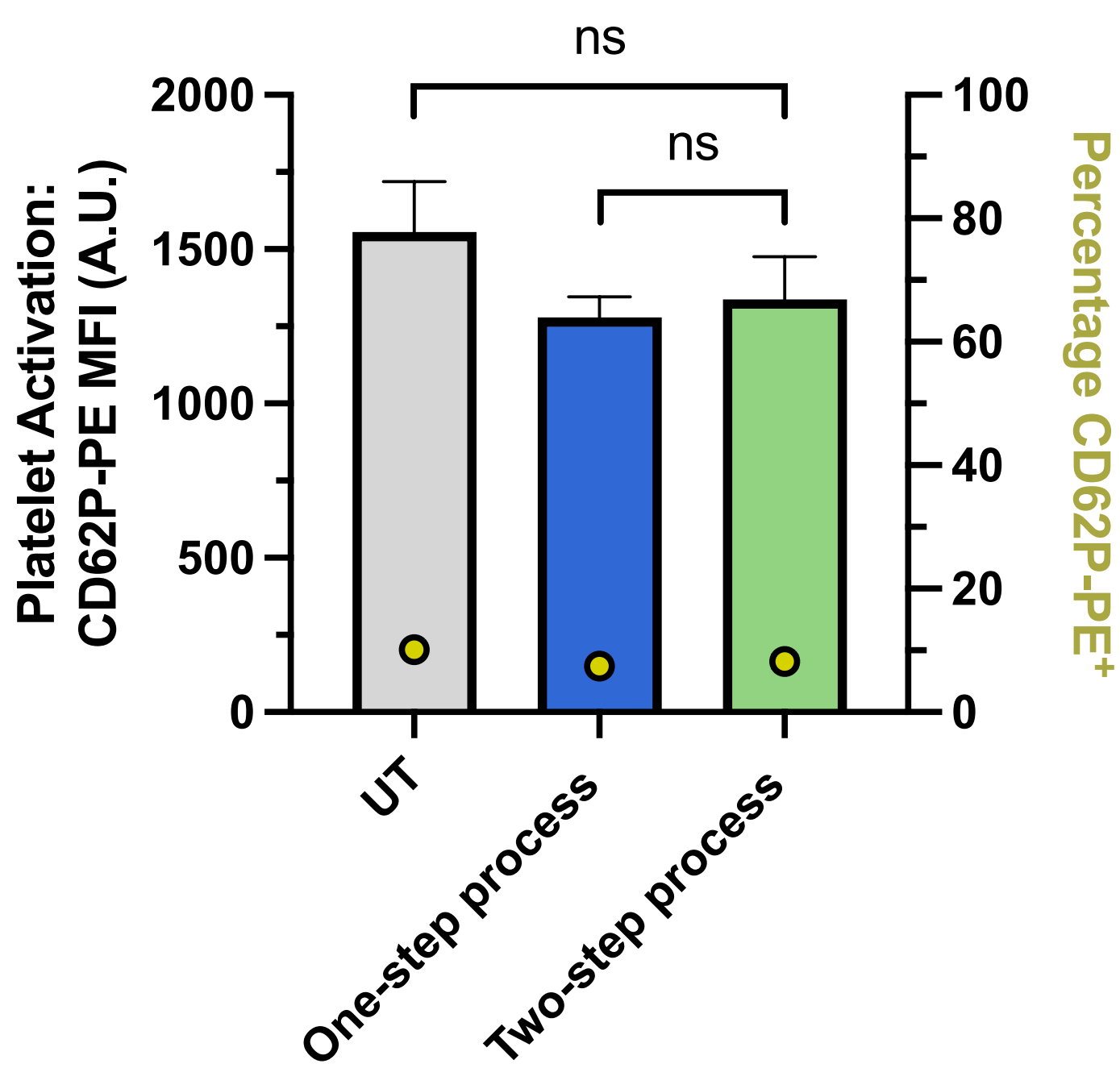

Figure S13. LNP mRNA formulated through the two-step buffer exchange process does not activate platelets. LNP mRNA (nLuc; N/P = 6) were formulated with a lipid composition consisting of NTX-001/POPC/Cholesterol/PEG-DMG (50/10/38.5/1.5 mol%, respectively) and were subjected to a one-step (PBS dialysis) or two-step (163 mM NaOAc + 25% EtOH dialysis prior to PBS dialysis) buffer exchange. Platelets were transfected at a concentration of 250 x 10^6 platelets/mL, and at an mRNA dose of 12 μg/mL. Platelet activation was measured with flow cytometry at 4 h post-treatment via the cell surface marker P-selectin (CD62P) conjugated to the fluorophore phycoerythrin (PE). Quantification of CD62P-PE mean fluorescence intensity (bars, left y axis) and percentage of platelets positive for CD62P-PE (yellow circles, right y axis) (mean ± S.E.M., n = 3). UT=untreated. Data was analyzed by a two-way ANOVA, ns = non-significant.

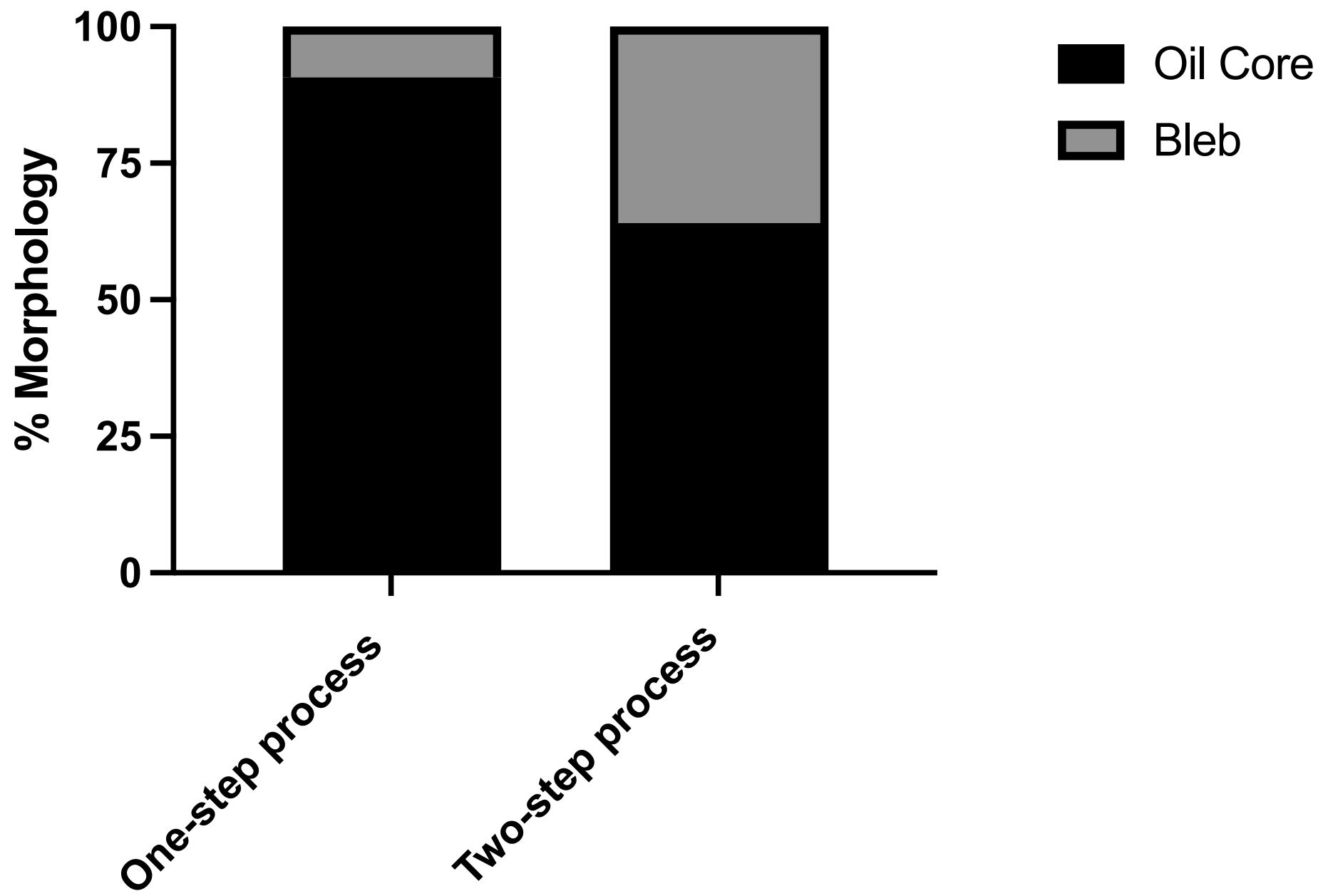

Figure S14. NTX-001 LNP mRNA formulations display a higher proportion of bleb morphology when manufactured using the two-step buffer exchange process. LNP mRNA (nLuc; N/P = 6) were formulated with a lipid composition consisting of NTX-001/POPC/Cholesterol/PEG-DMG (50/10/38.5/1.5 mol%, respectively) and were subjected to a one-step (PBS dialysis) or two-step (163 mM NaOAc + 25% EtOH dialysis prior to PBS dialysis) buffer exchange. The LNP morphologies from cryo-TEM micrographs were then classified into two categories (Oil Core or Bleb). Cryo-TEM morphology was manually classified (n = 300 particles).

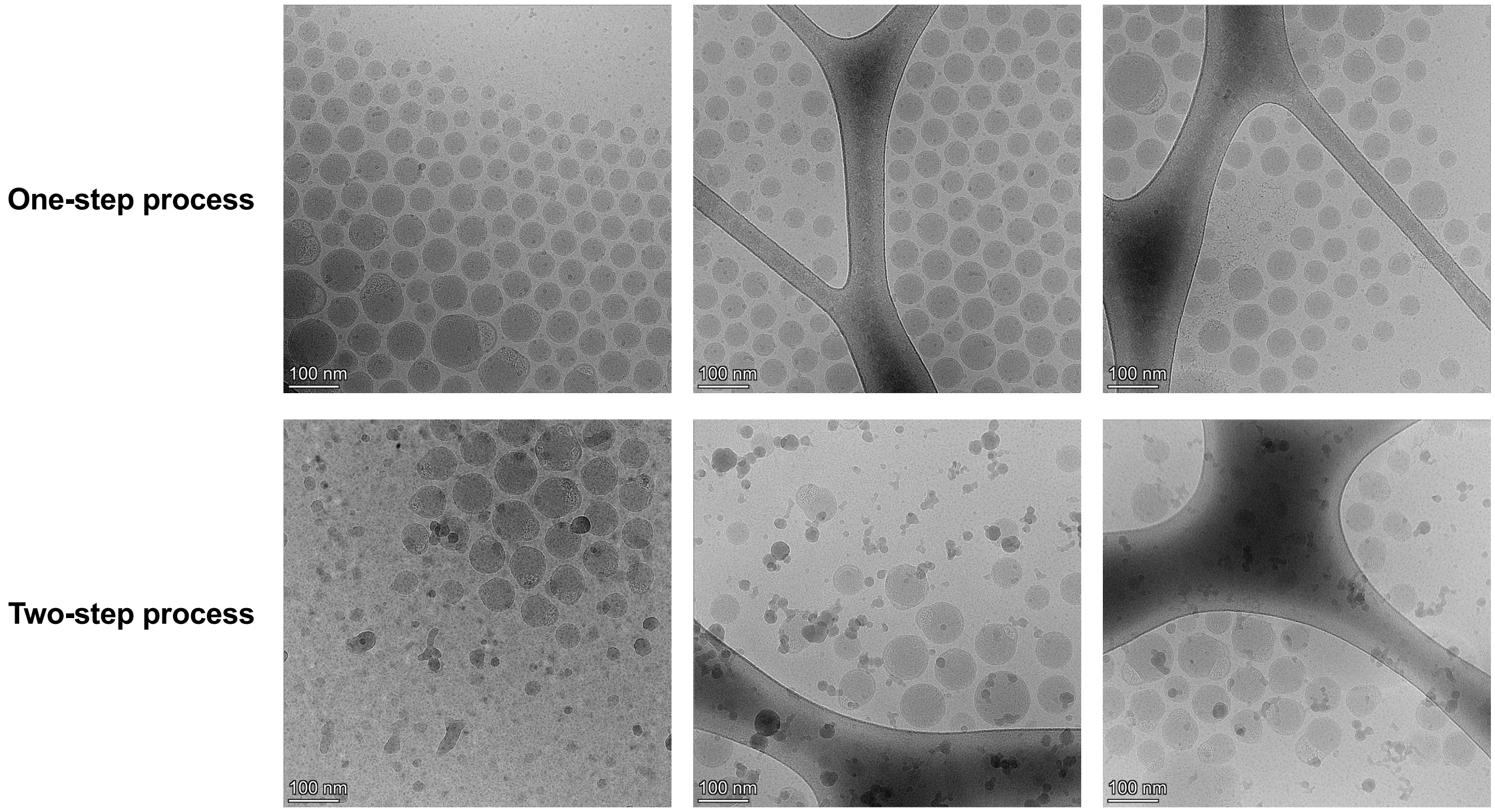

Figure S15. Additional representative cryo-TEM micrographs of NTX-001 LNP mRNA formulations. LNP mRNA (nLuc; N/P = 6) were formulated with a lipid composition consisting of NTX-001/POPC/Cholesterol/PEG-DMG (50/10/38.5/1.5 mol%, respectively) and were subjected to a one-step (PBS dialysis) or two-step (163 mM NaOAc + 25% EtOH dialysis prior to PBS dialysis) buffer exchange. The dispersion was then concentrated and imaged.

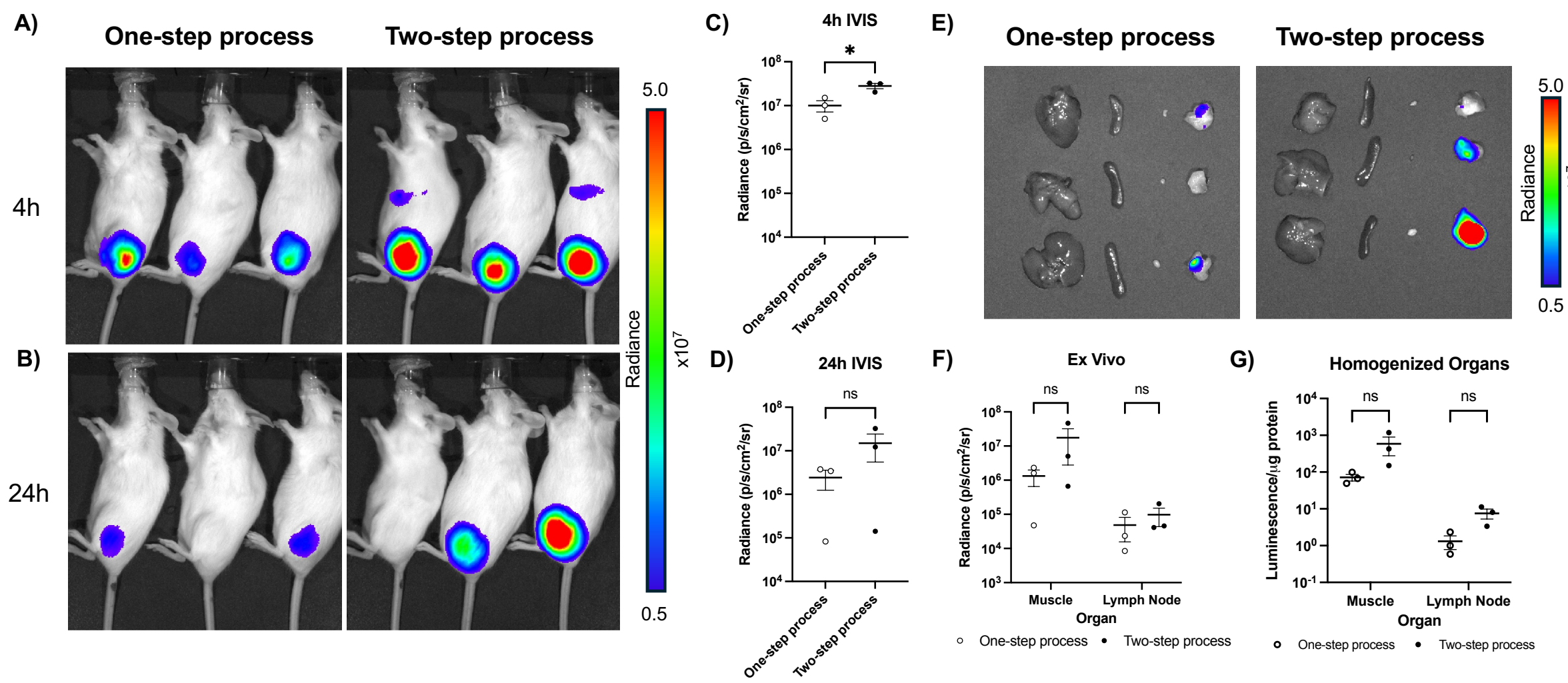

Figure S16. LNP mRNA produced from the two-step buffer exchange process slightly improves *in vivo* transfection following i.m. administration compared to LNP mRNA produced from a one-step buffer exchange. LNP mRNA (FLuc; N/P = 6) were formulated with an Onpattro-based composition consisting of norMC3/DSPC/Cholesterol/PEG-DMG (50/10/38.5/1.5 mol%, respectively) and were subjected to a one-step (PBS dialysis) or two-step (163 mM NaOAc + 25% EtOH dialysis prior to PBS dialysis) buffer exchange. LNP mRNA were administered i.m. at an mRNA dose of 0.1 mg/kg to CD-1 mice. **A–D)** IVIS bioluminescence imaging was performed at A) 4 h and B) 24 h post-injection with the corresponding radiance shown in C) and D), respectively (mean ± S.E.M., n = 3). **E)** *Ex vivo* bioluminescence in liver, spleen, inguinal lymph node, and muscle. **F)** *Ex vivo* radiance quantification of muscle and inguinal lymph node (mean ± S.E.M., n = 3) **G)** Homogenized muscle and lymph node bioluminescence was assayed through a luciferase assay and normalized by protein content (mean ± S.E.M., n = 3). All data were analyzed through a two-tailed unpaired t-test, ns = non-significant, *$p < 0.05$.

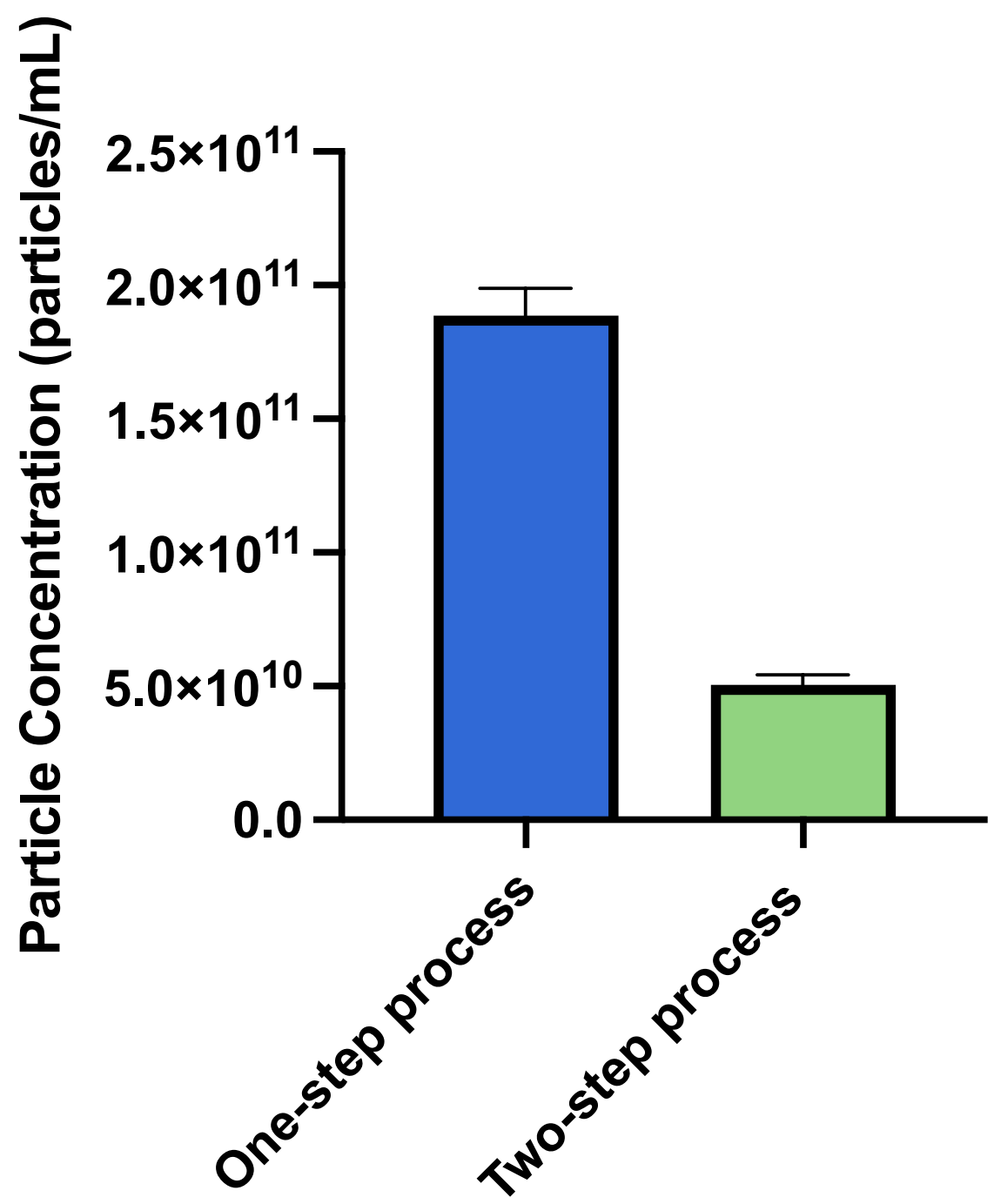

Figure S17. LNP mRNA formulations produced from the two-step buffer exchange process results in decreased particle concentrations compared to LNP mRNA produced from a one-step buffer exchange. LNP mRNA (FLuc; N/P = 6) were formulated with an Onpattro-based composition consisting of norMC3/DSPC/Cholesterol/PEG-DMG (50/10/38.5/1.5 mol%, respectively) and were subjected to a one-step (PBS dialysis) or two-step (163 mM NaOAc + 25% EtOH dialysis prior to PBS dialysis) buffer exchange. LNPs were diluted to 20 μg/mL total lipids and particle concentration was measured through DLS.

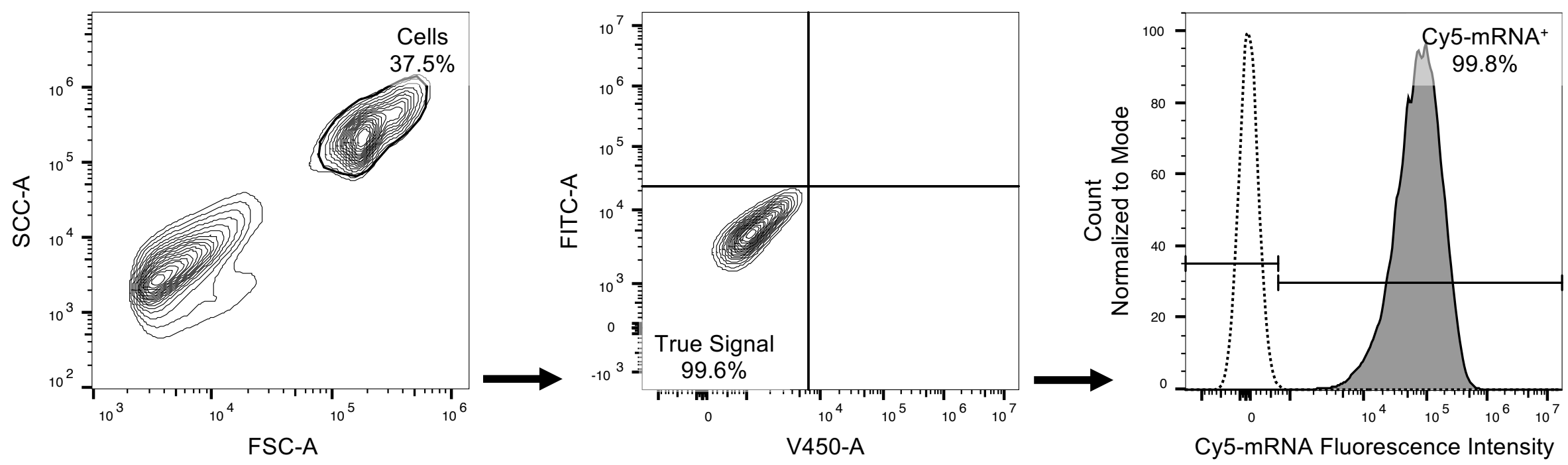

Figure S18. Representative flow cytometry gating scheme to evaluate LNP uptake in Huh7 cells. Viable cells were first gated based on their forward scatter (FSC-A) and side scatter (SSC-A) intensities[1–3], then gated further against the negative fluorescence channels, V450-A and FITC-A, to exclude autofluorescent events. Huh7 cells positive for Cy5-mRNA were then gated relative to the untreated control, represented as the population in the dotted line.

References

(1) Darzynkiewicz, Z.; Li, X.; Gong, J. Chapter 2 Assays of Cell Viability: Discrimination of Cells Dying by Apoptosis. In *Methods in Cell Biology*; Elsevier, 1994; Vol. 41, pp 15–38. https://doi.org/10.1016/S0091-679X(08)61707-0.
(2) Al-Rubeai, M.; Welzenbach, K.; Lloyd, D. R.; Emery, A. N. A Rapid Method for Evaluation of Cell Number and Viability by Flow Cytometry.
(3) Jiang, L.; Tixeira, R.; Caruso, S.; Atkin-Smith, G. K.; Baxter, A. A.; Paone, S.; Hulett, M. D.; Poon, I. K. H. Monitoring the Progression of Cell Death and the Disassembly of Dying Cells by Flow Cytometry. *Nat. Protoc.* **2016**, *11* (4), 655–663. https://doi.org/10.1038/nprot.2016.028.
